# Amphiregulin couples IL1RL1^+^ regulatory T cells and cancer-associated fibroblasts to impede antitumor immunity

**DOI:** 10.1126/sciadv.add7399

**Published:** 2023-08-23

**Authors:** Runzi Sun, Hongyu Zhao, David Shihong Gao, Andrew Ni, Haochen Li, Lujia Chen, Xinghua Lu, Kong Chen, Binfeng Lu

**Affiliations:** ^1^ Department of Immunology, University of Pittsburgh School of Medicine, Pittsburgh, PA, USA.; ^2^ Center for Discovery and Innovation, Hackensack Meridian Health, Nutley, NJ, USA.; ^3^ Department of Biomedical informatics, University of Pittsburgh School of Medicine, Pittsburgh, PA, USA.; ^4^ Department of Pulmonary, Allergy, and Critical Care Medicine, University of Pittsburgh School of Medicine, Pittsburgh, PA, USA.

## Abstract

Regulatory T (T_reg_) cells and cancer-associated fibroblasts (CAFs) jointly promote tumor immune tolerance and tumorigenesis. The molecular apparatus that drives T_reg_ cell and CAF coordination in the tumor microenvironment (TME) remains elusive. Interleukin 33 (IL-33) has been shown to enhance fibrosis and IL1RL1^+^ T_reg_ cell accumulation during tumorigenesis and tissue repair. We demonstrated that IL1RL1 signaling in T_reg_ cells greatly dampened the antitumor activity of both IL-33 and PD-1 blockade. Whole tumor single-cell RNA sequencing (scRNA-seq) analysis and blockade experiments revealed that the amphiregulin (AREG)–epidermal growth factor receptor (EGFR) axis mediated cross-talk between IL1RL1^+^ T_reg_ cells and CAFs. We further demonstrated that the AREG/EGFR axis enables T_reg_ cells to promote a profibrotic and immunosuppressive functional state of CAFs. Moreover, AREG mAbs and IL-33 concertedly inhibited tumor growth. Our study reveals a previously unidentified AREG/EGFR-mediated T_reg_/CAF coupling that controls the bifurcation of fibroblast functional states and is a critical barrier for cancer immunotherapy.

## Teaser

AREG/EGFR-mediated Treg/CAF coupling controls bifurcation of CAF and is a critical barrier for cancer immunotherapy.

## INTRODUCTION

The cytokine interleukin-33 (IL-33) is a danger-associated molecular pattern that alerts the immune system upon tissue stress or damage (*1*). Under homeostatic conditions, the inactive form of IL-33 is constitutively expressed in the nucleus of epithelial cells, endothelial cells, and fibroblasts, but environmental insult and tissue damage trigger the release of the “alarmin” into the surrounding tissue environment (*1*). IL-33 acts on the multitude of cells that express the IL-33 receptor, IL1RL1 (also called ST2), which include type 1, type 2, and regulatory T lymphocytes, macrophages, mast cells, basophils, dendritic cells (DCs), group 2 innate lymphoid cells (ILC2s), eosinophils, as well as fibroblasts. Through various target cells, IL-33 drives multiple immunological and pathological processes in asthma, allergy, infections, heart diseases, fibrotic diseases, graft-versus-host disease, obesity, and cancer [reviewed in (*2*, *3*)].

Recent studies have used IL-33 as a cancer immunotherapeutic to bolster type 1 immune responses. The basis for these therapies is that IL-33 is markedly down-regulated in high-grade cancers, which might serve as a mechanism to evade antitumor immunity (*4*, *5*). In addition, immune checkpoint inhibitors (ICIs)—which remove the “brakes” of antitumor immunity—require IL-33 in the tumor to achieve therapeutic efficacy (*6*–*8*). Furthermore, many studies have shown that treatment with IL-33 or overexpression of IL-33 in tumor cells inhibits tumor progression through boosting type 1 antitumor immune responses in vivo (*9*–*12*). Combination of IL-33 with ICIs produces additive type 1 immune responses and antitumor efficacy (*6*, *12*).

In addition to promoting immune responses, IL-33 expands a population of IL1RL1^+^ “tissue” T regulatory cells (*13*–*17*). IL1RL1^+^ T_reg_ cells were initially thought to play immunosuppressive and anti-inflammatory roles (*13*, *18*–*21*). However, recent studies have demonstrated that the IL1RL1^+^ T_reg_ cells are directly involved in nonimmune regulatory roles in tissue repair and maintaining barrier tissue integrity (*17*, *22*–*24*). The tissue remodeling capacity of IL1RL1^+^ T_reg_ cells has shown to be mediated by the expression of epidermal growth factor receptor (EGFR) ligand amphiregulin (AREG), which promotes the development and homeostasis of various organs, including the mammary, ovary glands, and visceral adipose tissue (VAT), and tissue repair following infection by a myriad of pathogens [reviewed in (*25*, *26*)]. The presence of IL1RL1^+^ T_reg_ cells in the tumor promotes tumor progression (*16*, *27*–*32*). Yet, the mechanisms—specifically the target cell types and molecular interactions—used by IL1RL1^+^ T_reg_ cells to alter the tumor microenvironment (TME) and promote tumorigenesis remain unknown.

It is well established that IL-33 promotes fibrosis in many pathological settings such as liver fibrosis, pancreatitis, kidney diseases, rheumatoid arthritis, and asthma (*33*–*37*). In addition, it has been demonstrated that cancer-associated fibroblast (CAF)–derived IL-33 directly promotes tumorigenesis and metastasis (*38*, *39*). CAFs have been shown to suppress immune responses through promoting T_reg_ cell accumulation in human cancer tissues (*40*, *41*). Both costimulatory molecules and immune checkpoint molecules have been shown to directly mediate the interaction between CAFs and T_reg_ cells (*41*). Notably, IL-33 produced by stromal cells has been involved in cross-talk between IL1RL1^+^ T_reg_ cells and mesenchymal cells in the VAT (*42*, *43*). These findings raise the question of whether IL-33 promotes cross-talk between T_regs_ and CAFs and thereby enhances T_reg_-mediated immune suppression in the TME.

In this study, we aimed to further dissect the IL-33–driven immune cellular network in tumors. We used paired single-cell RNA and T cell receptor (TCR) sequencing (scRNA-seq and scTCR-seq) to reveal the cellular composition and genetic programming of T lymphocytes in tumors that expressed a high level of IL-33. We further investigated how IL1RL1 signaling in T_reg_ cells affected IL-33–mediated antitumor immunity. Last, we focused on determining whether the AREG/EGFR axis is involved in mediating T_reg_ cell/CAFs cross-talk in the TME and its significance in IL-33–mediated antitumor immunity.

## RESULTS

### IL-33 drives robust CD8^+^ T cell responses

To study how IL-33 shapes immune responses in the tumor, we used a transplant mouse model of an engineered B16 melanoma cell line that overexpressed the secreted form of IL-33 (B16–IL-33) (*10*). Consistent with previous reports, overexpression of IL-33 significantly inhibited tumor growth and the antitumor efficacy of IL-33 required host IL-33 receptor IL1RL1 (fig. S1, A and B) (*10*). We performed paired scRNA-seq and scTCR-seq of TCR-β^+^ T cells from control B16 and B16–IL-33 tumors on day 9 post-tumor inoculation (fig. S1C). Our data consisted of 11,022 cells that belonged to three main T cell lineage populations: CD4^+^Foxp3^−^ conventional T (T_conv_) cells, CD4^+^Foxp3^+^ T_reg_ cells, and CD8^+^ T cells (fig. S1, D to F). We found increased accumulation of T_reg_ cells and CD8^+^ T cells and decreased accumulation of T_conv_ cells in B16–IL-33 compared to B16 tumors (fig. S1G).

**Fig. 1.**
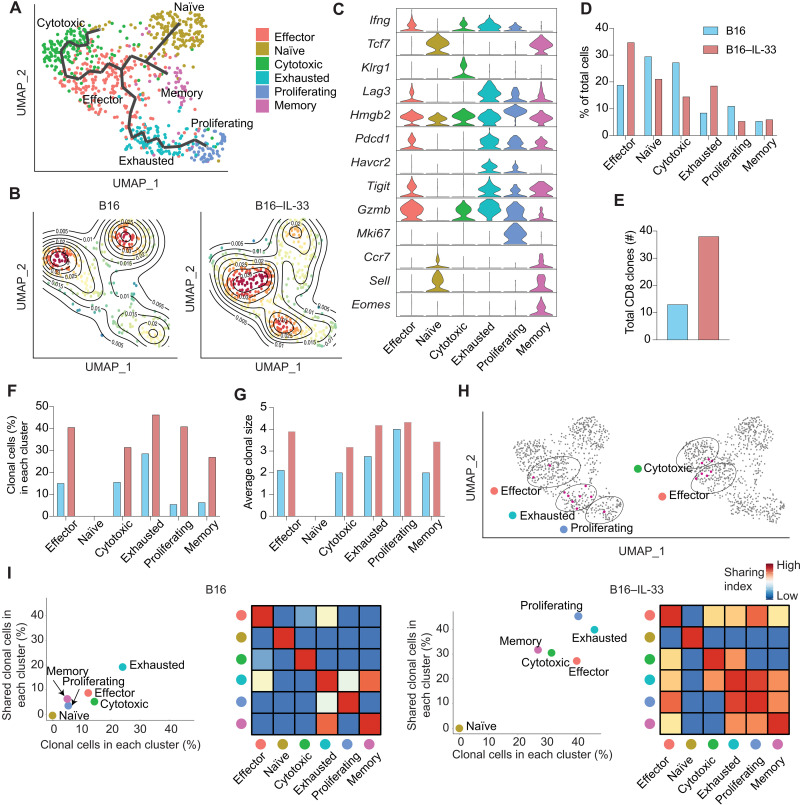
scRNA-seq analysis of CD8^+^ T cells in the TME of B16 and B16–IL-33. (**A**) UMAP dimensionality reduction projects CD8^+^ T cells from B16 and B16–IL-33 tumors to two dimensions showing six subclusters differentiated by color and trajectory analysis of different CD8 clusters. Each point represents a single cell, with cells of similar gene expression profiles positioned closer together in the projection. (**B**) Density plot for CD8^+^ T cells in the B16 (left) and B16–IL-33 (right) conditions. (**C**) Violin plot shows the expression of the top marker gene for all six clusters based on adjusted *P* value and log_2_ fold change (log_2_FC). (**D**) Bar plot showing the percentage of cells in each CD8^+^ T cell cluster based on tumor origin (B16 versus B16–IL-33). (**E**) Bar plot showing the total unique TCR clones in all CD8 clusters from B16 or B16–IL-33. (**F**) Bar plot showing the percentage of clonally expanded cells in each CD8^+^ T cell cluster. (**G**) Bar plot showing the average clonal size in each CD8^+^ T cell cluster. (**H**) Distribution of two representative CD8 clones on the UMAP plot. (**I**) Scatter plot comparing the percent of clonal cells (*x*) and percent of shared clonal cells in each cluster (*y*). The heatmap in the right panel depicts the percent of clonal cells shared between clusters.

We first analyzed whether there were any transcriptional differences between CD8^+^ T cells in B16–IL-33 versus B16 tumors. CD8^+^ tumor-infiltrating lymphocytes (TILs) could be grouped into six clusters that corresponded to naïve, memory, effector, cytotoxic, exhausted, and proliferating clusters (Fig. 1, A to C, and fig. S2, A and B). The trajectory analysis revealed that differentiation of the CD8^+^ TILs consisted of three paths: naïve → memory, naïve → effector → cytotoxic, and naïve → effector → exhausted → proliferative (Fig. 1A). We also grouped the clusters using hierarchical clustering based on the transcriptional programs of the cells in each cluster (fig. S2A). The clusters belonging to each path could be grouped together. Naïve and memory CD8^+^ TILs expressed *Tcf-7*, *Sell*, and *Ccr7*. Effector and cytotoxic cells expressed *Ifng* and *GzmB*. Exhausted and proliferative cells expressed immune checkpoint molecules, such as *Pdcd1*, *Havcr2*, *Tigit*, and *Lag3* (Fig. 1C and fig. S2B).

The analysis further showed that IL-33 led to a marked change in the composition of the CD8^+^ T cell subsets. B16–IL-33 tumors had an increased percentage of cells in the effector and exhausted clusters (Fig. 1, B and D). These two clusters highly expressed *Ifng*, the CD8^+^ T cell effector cytokine. We next performed flow cytometry to see whether production of the interferon-γ (IFN-γ) protein was increased in CD8^+^ TILs from B16–IL-33 tumors. Consistently, we found either treatment with IL-33 or overexpression of IL-33 in tumor cells significantly increased the percentage of IFN-γ^+^ CD8^+^ T cells in the TME (fig. S2, C and D).

We next examined clonal expansion in various CD8^+^ TIL subsets using the paired scTCR-seq data. The naïve population consisted of only single TCR clones in both B16 and B16–IL-33 tumors. In each remaining cluster, CD8^+^ TILs in B16–IL-33 tumors had a higher percentage of clonally expanded cells and a larger average clonal size (Fig. 1, F and G) than that of in B16 tumor. These data indicate that IL-33 caused marked clonal expansion of CD8^+^ TILs. The total number of clones was higher in B16–IL-33 tumors compared to B16 tumors, indicating that IL-33 also increases the clonal diversity of CD8^+^ TILs (Fig. 1E). These data indicated that IL-33 led to clonal expansion of CD8^+^ TILs.

Last, we studied whether clonally expanded CD8^+^ T cells differentiated into multiple clusters, which is evidence for functional diversification. We investigated the expanded clones projected on the Uniform Manifold Approximation and Projection (UMAP) to study the differentiation trajectory of individual T cell clones. One representative clone is present in all the clusters of the effector → exhausted → proliferating path, and another clone took an effector → cytotoxic path (Fig. 1H). We then studied all the expanded clones collectively. In B16 tumors, 20% of clonally expanded cells from the exhausted population had clones present in the other clusters, mostly the proliferating cluster (Fig. 1I, left panels). Cells in all other clusters had little clonal expansion and did not share clones with other clusters. In contrast, B16–IL-33 tumors, in all clusters—except for the naïve cluster—had more than 25% of clonally expanded cells shared with other clusters (Fig. 1I, right panels, and fig. S2F). Collectively, our scRNA-seq data show that overexpression of IL-33 in B16 tumors drives robust accumulation, clonal expansion, and functional diversification of CD8^+^ TILs.

### IL-33 increases accumulation of IL1RL1^+^ T_reg_ cells

**Fig. 2.**
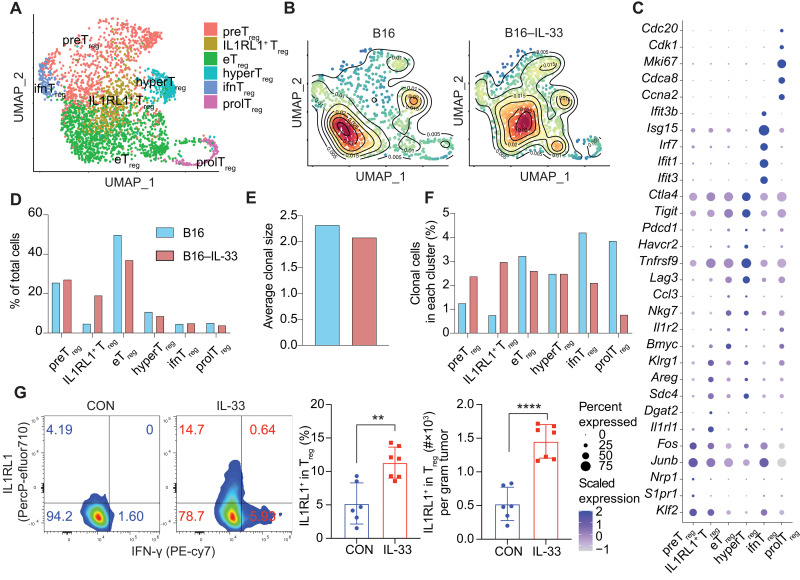
IL1RL1^+^ T_reg_ cells were greatly increased in the IL-33–expressing tumors. (**A**) UMAP dimensionality reduction projects T_reg_ cells from B16 and B16–IL-33 tumors to two dimensions showing six subclusters differentiated by color. Each point represents a single cell, with cells of similar gene expression profiles positioned closer together in the projection. (**B**) Density plot for T_reg_ cells in the B16 (left) and B16–IL-33 (right) conditions. (**C**) Dot plot shows the expression of the top marker gene for all six clusters based on adjusted *P* value and log_2_FC. (**D**) Bar plot showing the percentage of cells in each T_reg_ cell cluster based on tumor origin (B16 versus B16–IL-33). (**E**) Bar plot showing the average clonal size in T_reg_ cells in B16 or B16–IL-33 tumors. (**F**) Bar plot showing the percentage of clonally expanded cells in each T_reg_ cell cluster. (**G**) MC38 tumor cells (1 × 10^6^) were inoculated intradermally into the right flank of the C57BL/6J mice. IL-33 protein or PBS was adminstered starting from day 5 and again every 4 days for a total of three times. Representative flow cytometry plot showing ST2 staining gated on T_reg_ cells 17 days post-tumor inoculation. Bar plot showing the percentage and number of ST2^+^ T_reg_ cells. Data are representative of three independent experiments in (G). Bar graphs represent data summarized as means ± SEM. **P < 0.01 and ****P < 0.0001. PE, phycoerythrin.

We used our scRNA-seq data to examine T_reg_ cells from B16 and B16–IL-33 tumors. The cells formed six clusters corresponding to pre-effector (preT_reg_), IL1RL1 (IL1RL1^+^ T_reg_), effector (eT_reg_), hyperactivated effector (hyperT_reg_), interferon-signature (ifnT_reg_), and proliferating (prolT_reg_) T_regs_ (Fig. 2, A to C). preT_regs_ expressed early activation genes, including *Fos*, *Jun*, and *Klf2*. eT_regs_ and hyperT_regs_ expressed immune checkpoint molecules, including *Pdcd1*, *Havcr2*, *Tigit*, *Lag3*, and *Ctla4*, as well as the costimulatory molecule *Tnfrsf9*. IfnT_regs_ expressed genes that typically induced by interferons such as *Irf7*, *Ifit1*, *Ifit3*, and *Isg15*, and prolT_regs_ expressed genes that are involved in cell proliferation such as *Mki67* and *Cdk1*. IL1RL1^+^ T_reg_ cells expressed a unique set of marker genes including *Il1rl1*, *Areg*, *Klrg1*, and *Sdc4* (Fig. 2C).

We found a notable increase in the accumulation of IL1RL1^+^ T_regs_ in B16-lL33 compared to B16 tumors (Fig. 2B and fig. S1G). We also found that B16–IL-33 tumors had a decrease in the percentage of eT_reg_ cells (Fig. 2D). We also analyzed clonal expansion using our paired scTCR-seq data. B16–IL-33 tumors had a higher percentage of preT_reg_ and IL1RL1^+^ T_reg_ cells that were clonally expanded (Fig. 2F). In contrast, the percentages of clonally expanded eT_reg_, ifnT_reg_, and prolT_reg_ cells were greater in B16 tumors (Fig. 2F). The average clonal size of T_reg_ cells from B16 and B16–IL-33 tumors was about the same (Fig. 2E). We further confirmed our results using another transplant mouse model of MC38 colon carcinoma using flow cytometry. Treatment of MC38 tumors with four rounds of IL-33 increased the accumulation of IL1RL1^+^ T_reg_ cells in the tumor (Fig. 2G). Together, these data indicated that IL-33 markedly increases the accumulation and clonal expansion of IL1RL1^+^ T_reg_ cells in tumors.

### Deletion of *Il1rl1* on T_regs_ enhances antitumor immunity

**Fig. 3.**
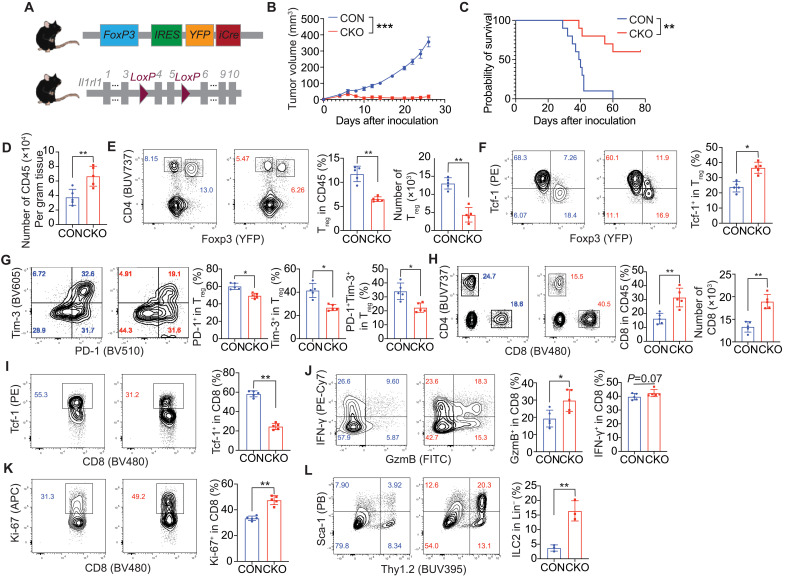
Specific deletion of on *Il1rl1* in T_regs_ altered lymphocyte population in the TME. (**A**) strategy of generation of *Foxp3*^cre^*Il1rl1*^flox/flox^ mice. (**B** and **C**). B16–IL-33 tumor cells (1 × 10^5^) were inoculated intradermally into the right flank of the female CON (*Foxp3*^cre/cre^) and CKO (*Foxp3*^cre/cre^*Il1rl1*^flox/flox^) mice. Tumor size was monitored every 2 days. Tumor curve (B) and overall survival (C) of B16–IL-33 tumor-bearing mice. (**D**) Bar plot showing the number of CD45 cells per gram tumor tissue in B16–IL-33 tumor-bearing CON and CKO mice. (**E**) Representative flow cytometry plot and a quantitative plot showing the percentage and number of T_reg_ cells. (**F**) Representative flow cytometry plot and a quantitative plot showing the percentage of Tcf-1^+^ T_reg_ cells. (**G**) Representative flow cytometry plot and quantitative plot of the percentage of PD-1^+^, Tim-3^+^, or PD-1^+^Tim-3^+^ T_reg_ cells. (**H**) Representative flow cytometry plot and quantitative plot showing the percentage and number of CD8^+^ T cells. (**I**) Representative flow cytometry plot and quantitative plot showing the percentage of Tcf-1^+^ CD8^+^ T cells. (**J**) Representative flow cytometry plot and quantitative plot showing the percentage of GzmB^+^ CD8^+^ T cells and IFN-γ^+^ CD8^+^ T cells. (**K**) Representative flow cytometry plot and quantitative plot showing the percentage of Ki-67^+^ CD8^+^ T cells. (**L**) Representative flow cytometry plot and quantitative plot showing the percentage of ILC2 cells. Day 13 tumors were analyzed in (D), and day 8 tumors were analyzed in (E) to (I). Data shown are representative of three to five independent experiments. Graphs shown represent data summarized as means ± SEM and were analyzed by unpaired two-tailed Student’s *t* test. Two-way analysis of variance (ANOVA) was used to determine statistical significance for time points when all mice were viable for tumor measurement. *P < 0.05, **P < 0.01, and ***P < 0.001. APC, allophycocyanin; PB, Pacific Blue; YFP, yellow fluorescent protein; IRES, internal ribosomal entry site; FITC, fluorescein isothiocyanate.

To investigate the role of IL1RL1 signaling on T_reg_ cells in regulating IL-33–mediated antitumor immune responses, we generated *Foxp3^cre^Il1rl1^Wt/Wt^* (CON) and *Foxp3^cre^Il1rl1^Flox/Flox^* (CKO) mice (Fig. 3A and fig. S3, A and B). The CKO mice showed normal T cell development and homeostasis (fig. S3, C and D). Inoculation of B16–IL-33 cells showed that tumor growth was significantly decreased in CKO versus CON mice (Fig. 3B and fig. S3E). About 67% (six of nine) of the CKO mice became tumor-free, which was the case for none of the CON mice. In addition, the overall survival was significantly prolonged in the CKO mice (Fig. 3C and fig. S3F). We also treated CKO mice with MC38 tumors using programmed cell death protein 1 (PD-1) monoclonal antibodies (mAbs). CKO mice again had a significant decrease in tumor growth in the treatment condition (fig. S3G). For the untreated group, the tumors grew at a comparable rate between the CON and CKO mice.

We next tested whether IL1RL1 on T_reg_ cells affected immune responses in the tumor. To this end, we performed multicolor flow cytometry on single-cell suspensions of B16–IL-33 tumors from CON and CKO mice. We assayed tumors around day 8 when the first difference in volume between the conditions was observed. Tumors from CKO mice had increased accumulation of total CD45^+^ immune cells (Fig. 3D). We next looked at how IL1RL1 deficiency regulated the accumulation and activation of T_reg_ cells in the tumor. The accumulation of CKO T_regs_ was markedly reduced compared to CON T_regs_ (Fig. 3E). It was reported that T cell factor 1 (Tcf-1) impaired T_reg_ generation and immunosuppressive capacity (*44*, *45*). CKO T_regs_ had a more naïve-like phenotype (Tcf-1^+^) (Fig. 3F). PD-1 and T cell immunoglobulin and mucin domain-containing protein 3 (Tim-3) are characteristically expressed on effector T_regs_ and are associated with increased suppressor function (*46*). We found a decrease in the percentage of PD-1^+^, Tim-3^+^, and PD-1^+^Tim-3^+^ T_reg_ cells in the CKO condition (Fig. 3G).

We next determined whether IL1RL1 deficiency on T_regs_ led to further changes to CD8^+^ TILs. The percentage of CD8^+^ T cells in the B16–IL-33 tumors was increased in CKO compared to CON mice (Fig. 3H). The CKO condition had a lower percentage of resting cells (Tcf-1^+^) and a higher percentage of proliferative cells (Ki-67^+^) (Fig. 3, I and K). Moreover, the CKO condition had a higher percentage of cells that expressed Granzyme B (GzmB) and IFN-γ (Fig. 3J). This suggests that lack of IL1RL1 on T_reg_ cells promotes the effector function of CD8^+^ TIL. PD-1^+^Tim-3^+^ double-positive (DP) CD8^+^ TILs have been shown to be both hyperactivated effector cells and exhausted T cells in different studies (*46*–*49*). The percentage of PD-1^−^Tim-3^−^ double-negative (DN) and PD-1^+^Tim-3^−^ single-positive (SP) cells were decreased and unchanged, respectively, between the CON and CKO conditions (fig. S4A). However, the percentage of DP cells doubled in tumors from CKO mice (fig. S4A). The up-regulation of exhaustion markers of CD8^+^ TILs suggest enhanced tumor reactivity, since previous literature has shown that tumor-reactive CD8^+^ T cells become exhausted due to persistent tumor stimulation (*50*, *51*). The CKO condition also had a higher percentage of CD39^+^ PD-1^+^ cells (fig. S4B) (*51*, *52*). This suggests that lack of IL1RL1 on T_reg_ promotes hyperactivation and expression of inhibitory molecules on CD8^+^ TILs.

A recent study showed that ILC2s contribute to the antitumor efficacy of Il-33 treatment (*7*). Therefore, we checked whether ILC2s accumulated in B16–IL-33 tumors and if this phenomenon was affected by the presence of *Il1rl1* on T_regs_. We found that ILC2s accumulated in B16–IL-33 tumors from CON mice, and this accumulation was greatly increased in CKO mice (Fig. 3L). Together, our data show that deletion of *Il1rl1* on T_reg_ cells inhibited the growth of tumors containing Il-33. Tumors with IL1RL1-deficient T_reg_ cells have decreased accumulation of effector T_regs_, increased accumulation and activation of CD8^+^ TILs, and increased accumulation of ILC2s.

**Fig. 4.**
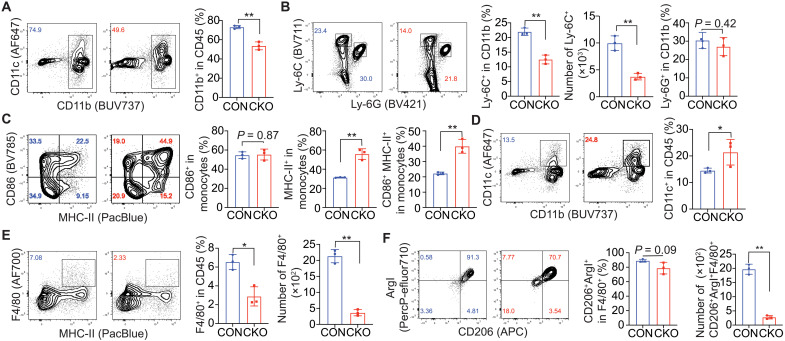
The changes of myeloid compartments in the TME upon deletion of Il1rl1 in T_reg_ cells. (**A**) Representative flow cytometry plot and quantitative plot showing the percentage CD11b^+^ cells in total CD45^+^ cells. (**B**) Representative flow cytometry plot and quantitative plot showing the percentage and number of monocytic MDSCs and granulocytic MDSCs. (**C**) Representative flow cytometry plot and quantitative plot of the percentage of CD86^+^, MHC-II^+^, or CD86^+^MHC-II ^+^ monocytes. (**D**) Representative flow cytometry plot and quantitative plot showing the percentage of DCs in total CD45^+^ cells. (**E**) Representative flow cytometry plot and quantitative plot showing the percentage and number of infiltrating macrophages. (**F**) Representative flow cytometry plot and quantitative plot showing the percentage and number of type 1 and type 2 macrophages. Data shown are representative of three to five independent experiments (day 8 takedown). Graphs shown represent data summarized as means ± SEM and were analyzed by unpaired two-tailed Student’s *t* test. *P < 0.05 and **P < 0.01.

We next investigated whether IL1RL1 deficiency on T_reg_ cells also resulted in changes to tumor-associated myeloid cells. The accumulation of CD11b^+^ myeloid cells in the B16–IL-33 tumors was decreased in CKO compared to CON mice (Fig. 4A). While the accumulation of granulocytic myeloid-derived suppressor cells (gMDSCs; Ly-6G^+^) was comparable between the two conditions, the accumulation of monocytic MDSCs (mMDSCs; Ly-6C^+^) was reduced in the CKO condition (Fig. 4B). The CKO condition had a higher percentage of mMDSCs that expressed major histocompatibility complex II (MHC-II) (Fig. 4C). Tumors from the CKO mice also had more accumulation of DCs (CD11b^+^CD11c^+^) (Fig. 4D). In particular, there was a higher percentage of type 1 DCs (DC1s; CD103^+^) (fig. S4D). Last, the accumulation of tumor-associated macrophages (TAMs; F4/80^+^MHC-II^+^) was decreased in tumors from CKO mice (Fig. 4E). The CKO condition had decreased accumulation of type 2 TAMs (CD206^+^Arg1^+^) (Fig. 4F). Our data showed that the deletion of IL1RL1 on T_reg_ cells shifted tumor-associated myeloid cells away from an immunosuppressive phenotype. Collectively, we showed that IL1RL1 deficiency on T_regs_ enhanced antitumor immunity and inhibited the growth of B16–IL-33 tumors.

### IL1RL1 is required for IL-33–driven accumulation of IL1RL1^+^ T_regs_ in the tumor

**Fig. 5.**
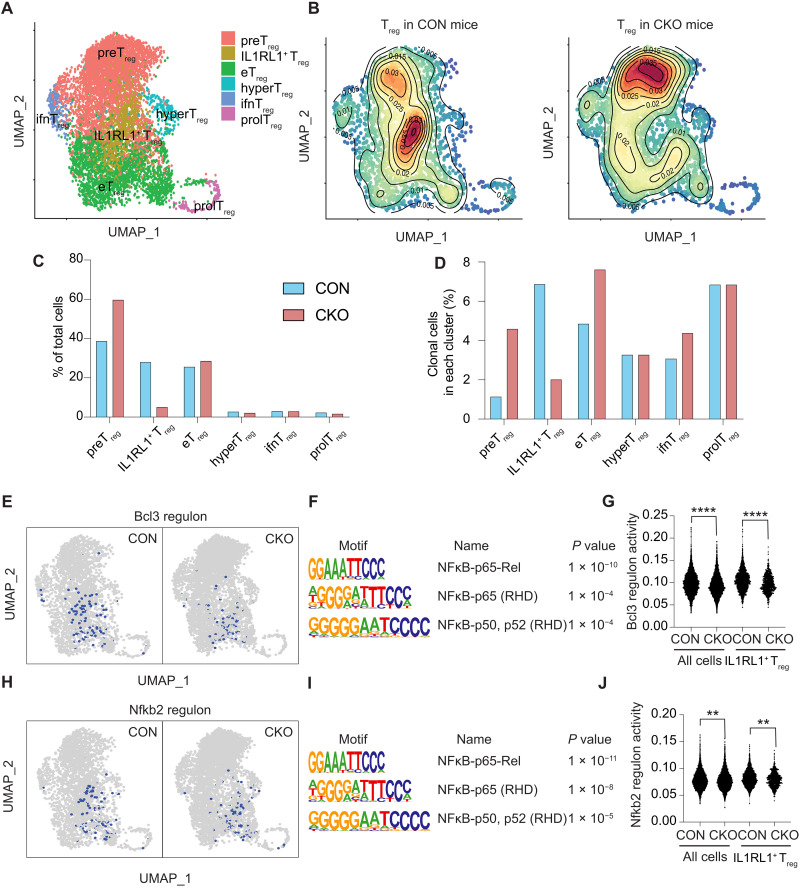
Global transcriptional landscape of T_reg_ cells from tumor-bearing CON or CKO mice. (**A**) UMAP dimensionality reduction projects T_reg_ cells from day 9 B16–IL-33 tumor-bearing CON or CKO mice to two dimensions showing six subclusters differentiated by color. Each point represents a single cell, with cells of similar gene expression profiles positioned closer together in the projection. (**B**) Density plot for T_reg_ cells in CON (left) or CKO (right) mice. (**C**) Bar plot showing the percentage of cells in each T_reg_ cell cluster based on the tumor origin (CON mice versus CKO mice). (**D**) Bar plot showing the percentage of clonally expanded cells in each T_reg_ cell cluster. (**E**) UMAP projection comparing the distribution of Bcl3 regulon in B16–IL-33 tumor-bearing CON or CKO mice. (**F**) Motif enriched in the promoter region of Bcl3 target genes generated by HOMER. (**G**) Violin plot showing the Bcl3 regulon activity of T_reg_ cells in all clusters and in IL1RL1^+^ T_reg_ cluster. (**H**) UMAP projection comparing the distribution of Nfkb2 regulon in B16–IL-33 tumor-bearing CON or CKO mice. (**I**) Motif enriched in the promoter region of Nfkb2 target genes generated by HOMER. (**J**) Violin plot showing the Nfkb2 regulon activity of T_reg_ cells in all clusters and in IL1RL1^+^ T_reg_ cluster. **P < 0.01 and ****P < 0.0001. RHD, Rel homology domain; NFκB, nuclear factor κB.

To study how the transcriptional program of T_regs_ was affected by IL1RL1, we performed scRNA-seq on CON and CKO T_regs_ from day 9 B16–IL-33 tumors. We identified the same T_reg_ clusters that were present in our first batch of scRNA-seq data (Figs. 5, A and B, and 2, A to C). The clusters were formed using the genome-wide transcriptional program of each cell. Therefore, we could identify cells that match IL1RL1^+^ T_regs_ even in the absence of IL1RL1. We found a notable decrease in the accumulation of CKO compared to CON IL1RL1^+^ T_regs_ (Fig. 5C). The CKO condition had increased accumulation of preT_regs_ and eT_regs_ (Fig. 5C). The percentage of clonally expanded cells was lower in IL1RL1^+^ T_regs_ from the CKO condition but higher in preTregs, eT_regs_, and ifnT_regs_ (Fig. 5D).

Next, we identified key transcription factors (TFs) that program IL1RL1^+^ T_regs_ from B16–IL-33 tumors. We used single-cell regulatory network inference and clustering [SCENIC; (*53*)] to find TFs and their target genes, which together are termed regulons. SCENIC calculates the regulon activity in each single cell based on the expression of TFs and target genes. We identified regulons of interest that exhibit differential activity in two ways: (i) IL1RL1^+^ T_reg_ cells versus the rest of T_reg_ subsets and (ii) CON versus CKO T_reg_ cells (*53*). The regulons with the highest activities were those of Bcl3 and Nfkb2 (Fig. 5, F to J). Both TFs regulate target genes through the nuclear factor κB sites (*54*, *55*). In addition, we also found that signal transducers and activators of transcription 1 (Stat1) and Maf regulons were specifically up-regulated in IL1RL1^+^ T_reg_ cells (fig. S5). Our data show that IL1RL1 is required for the accumulation of IL1RL1^+^ T_reg_ cells in B16–IL-33 tumors and that the transcriptome of IL1RL1^+^ T_reg_ cells is potentially regulated by Bcl3, Nfkb2, Stat1, and Maf.

### Coupling of IL1RL1^+^ T_regs_ and CAFs via an AREG/EGFR axis drives tumor immunosuppression

**Fig. 6.**
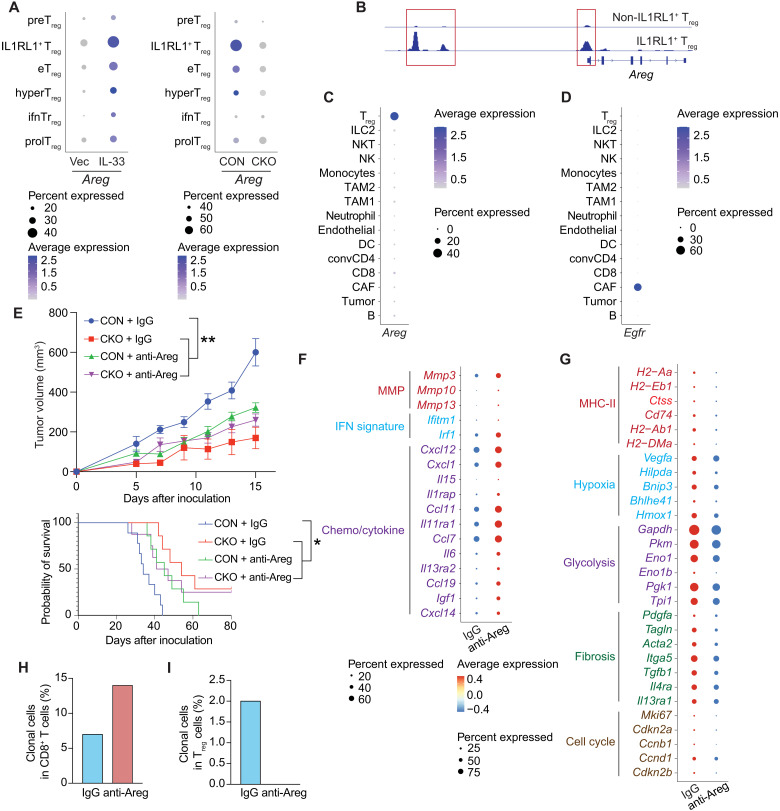
Areg/EGFR enabled IL1RL1^+^ T_regs_ and CAFs cross-talk to drive tumor immune suppression. (**A**) Dot plot showing the *Areg* gene expression across all T_reg_ clusters in B16 and B16–IL-33 tumors (left) and in B16–IL-33 tumor-bearing CON and CKO mice (right). (**B**) Genome track of *Areg* locus in lung tissue IL1RL1^+^ T_reg_ cells and other T_reg_ cells. (**C**) Dot plot showing the *Areg* gene expression level across all clusters in B16–IL-33 tumor microenvironment based on whole tumor scRNA-seq analysis. (**D**) Dot plot showing the *Egfr* gene expression level across all clusters in the B16–IL-33 tumor microenvironment based on whole tumor scRNA-seq analysis. (**E**) B16–IL-33 tumor cells (1 × 10^5^) were inoculated intradermally into the right flank of the CON and CKO mice. Anti-Areg antibody (50 μg per mouse) was treated starting from day 5 and every 4 days for a total of three times. Tumor growth curves and overall survival of B16–IL-33 tumor-bearing mice are shown. (**F**) Dot plot showing the up-regulated genes in CAFs from B16–IL-33 tumor-bearing mice treated after an anti-Areg antibody treatment based on whole tumor scRNA-seq analysis. (**G**) Dot plot showing the down-regulated genes in CAFs from B16–IL-33 tumor-bearing mice treated after an anti-Areg antibody treatment (day 9) based on whole tumor scRNA-seq analysis. (**H**) Bar plot showing the percentage of clonally expanded cells in CD8^+^ T cells based on whole tumor scRNA-seq analysis. (**I**) Bar plot showing the percentage of clonally expanded cells in T_reg_ cells based on whole tumor scRNA-seq analysis. Data shown are representative of three independent experiments. Graphs shown represent data summarized as means ± SEM and were analyzed by two-way ANOVA. **P < 0.01. NKT, natural killer T; DC, dendritic cell; TAM1, type 1 tumor-associated macrophage; convCD4, conventional CD4; IgG, immunoglobulin G.

To determine how IL1RL1^+^ T_reg_ cells suppress antitumor immune responses, we examined genes that are specifically up-regulated in IL1RL1^+^ T_reg_ cells and have an immune suppression function. To this end, we analyzed our two rounds of scRNA-seq data. *Areg* was up-regulated in IL1RL1^+^ T_reg_ cells versus other T_reg_ clusters (Figs. 2C and 6A). An independent analysis using a nested hierarchical Dirichlet process (nHDP) model also identified a gene expression module (GEM) that contained IL1RL1^+^ T_reg_ marker genes, which include Areg. CKO IL1RL1^+^ T_reg_ cells greatly down-regulated *Areg* compared to their CON counterparts (Fig. 6A). We also analyzed a published dataset for transposase-accessible chromatin sequencing (ATAC-seq) data of IL1RL1^+^ T_reg_ cells and other T_regs_ from lung tissue. IL1RL1^+^ T_reg_ cells had markedly increased chromatin accessibility at the *Areg* locus compared to other T_regs_ (Fig. 6B).

We next checked whether IL1RL1^+^ T_reg_ cells were the sole producers of *Areg*. scRNA-seq was performed on single-cell suspensions made from the following whole tumors: B16-, B16–IL-33–, and IL-33–treated MC38 from CON mice or CKO mice. Clustering using known marker genes identified the major cell types in the TME (figs. S6 and S7E). In B16 tumors, we found that *Areg* was exclusively expressed by T_reg_ cells (Fig. 6C). *Areg* was up-regulated in T_reg_ cells from B16–IL-33 versus B16 tumors (Fig. 6A and fig. S7, E and F). Consistently, Areg was also expressed almost exclusively on T_reg_ cells from the MC38 tumors (fig. S7, A and G). These data demonstrate that *Areg* is specifically regulated by IL-33 in T_reg_ cells. Notably, AREG expressed by T_reg_ cells has been shown to promote tumor growth (*25*, *26*). Therefore, it is possible that Areg can mediate the immune suppressive function of T_reg_ cells in the B16–IL-33 tumor.

To determine the target cells of IL1RL1^+^ T_reg_–derived AREG in the TME, we looked in our whole tumor scRNA-seq data to see which cell types expressed *Egfr*, the only known receptor for AREG (*56*). In all tumors, we found that EGFR was predominantly expressed in CAFs (Fig. 6D). EGFR was expressed by tumor cells as well, albeit generally at a much lower level than CAF. In addition, there was no evidence of EGFR expression in T cells, natural killer cells, myeloid cells, and other immune cell types in the TME. We also analyzed published whole tumor scRNA-seq data of murine B16 and pancreatic ductal adenocarcinoma. In both types of cancer, *Egfr* expression was mainly restricted to CAFs (fig. S7, C and D) (*57*, *58*). Our data suggested that IL1RL1^+^ T_reg_ cells could be coupled to CAFs via the AREG/EGFR axis.

To determine the role of Areg in mediating IL1RL1^+^ T_reg_ and CAF cross-talk and antitumor immunity, we treated B16–IL-33 tumor-bearing mice with an anti-AREG mAb. AREG mAb treatment significantly inhibited tumor growth and prolonged overall survival (Fig. 6E). However, we observed no additional therapeutic efficacy with anti-AREG antibodies in CKO mice, showing that Areg functions specifically from IL1RL1^+^ T_reg_ cells. We next asked whether the cross-talk between IL1RL1^+^ T_reg_ cells and CAFs altered the transcriptional program of CAFs. To this end, we performed scRNA-seq analysis of CAFs from day 9 B16–IL-33 tumors and those treated with AREG antibodies (Fig. 6, F and G). The data indicated that AREG blockade resulted in a decrease in genes in CAFs that promote fibrosis, proliferation, MHC-II antigen presentation, hypoxia, and glycolysis (Fig. 6G). In contrast, AREG antibodies increased genes encoding inflammatory cytokines, chemokines, and matrix metalloproteinases, as well as IFN-responsive gene signatures (Fig. 6F and fig. S8A). In addition, AREG blockade led to an increase in CD8^+^ T cell clonal expansion, a decrease in T_reg_ cell clonal expansion, and an increase of various inflammatory genes in the myeloid cells in the TME (Fig. 6, H and I, and fig. S8B). Collectively, our data showed that IL1RL1^+^ T_reg_ cells coupled with CAFs via an *AREG/EGFR* axis to promote tumor immunosuppression.

## DISCUSSION

The alarmin IL-33 potently not only stimulates immune responses in a variety of pathologic scenarios but also imposes regulatory checks and promotes tissue repair. By controlling a complex cellular network involving epithelial, immune, and stromal cells, IL-33 orchestrates a balanced response that ultimately returns the body to homeostasis. Here, we explored how IL-33 orchestrates the T lymphocyte response to cancer by using transplant mouse tumor models. IL-33–driven antitumor immunity—including accumulation, clonal expansion, and functional diversification of CD8^+^ TILs—was limited by the expansion of IL1RL1^+^ T_reg_ cells. AREG was specifically expressed in IL1RL1^+^ T_reg_ cells in IL-33–expressing tumors. We further demonstrated that AREG played a critical role in coupling IL1RL1^+^ T_reg_ cells to CAFs and thereby restricting the antitumor efficacy of IL-33. Mechanistically, in the presence of T_reg_-derived AREG, fibroblasts develop into a profibrotic functional state and facilitate tumor growth and immune suppression. In the absence of T_reg_-expressed AREG, fibroblasts develop to a proimmune state and mediate antitumor immune responses.

IL1RL1^+^ T_reg_ cells exert strong immunosuppressive and anti-inflammatory functions in wound healing, tissue homeostasis, autoimmunity, and cancer (*5*, *14*, *16*, *59*–*61*). IL1RL1^+^ T_reg_ cells play a major and nonredundant role in muscle and lung tissue repair after injury, independent with their role in immune suppression (*17*, *25*). AREG produced by IL1RL1^+^ T_reg_ cells has been shown to be an important effector molecule for tissue repair (*25*, *26*). Although AREG produced by mast cells and basophils have been found to regulate immune responses by promoting EGFR signaling in T_reg_ cells (*62*, *63*), in our experimental setting, we did not detect the expression of EGFR on T_reg_ cells. Nonetheless, we showed that AREG inhibited the antitumor efficacy of IL-33 treatment, indicating a protumor function of AREG in vivo. Our finding is consistent with a recent report showing that deletion of *Areg* in CD4^+^ T cells or T_reg_ cells inhibits lung cancer (*64*). In addition, we found that EGFR is specifically expressed in CAFs but not T_reg_ cells in the TME. These data support the idea that IL1RL1^+^ T_reg_ cells exert a protumor function through the AREG/EGFR-mediated interaction with CAFs. It has been shown that EGFR signaling on fibroblasts induces transcription of *Tgfb* (*65*, *66*). In addition, AREG has been shown to activate the TGF-β protein (*67*). Our scRNA-seq analysis also indicates that AREG promotes *Tgfb* expression in CAFs. Therefore, it is possible that AREG-induced TGF-β in CAFs mediates immunosuppression in the TME. Consistent with this idea, our data show that the blockade of AREG also greatly increased the level of mRNA of inflammatory cytokines in CAFs. It is worth mentioning that many immune cells were reported to express EGFR (*68*). EGFR was found to be expressed by T_reg_ cells, and the AREG-EGFR signaling axis is critical for maintaining Foxp3 stability in T_reg_ cells in patients with cancer (*62*, *63*). Therefore, future studies should be conducted to delete Egfr precisely in various cell types in different experimental settings to investigate the significance of EGFR signaling in establishing immune tolerance.

Emerging evidence supports the notion that CAFs enhance immunosuppression in the TME through direct interaction with T_reg_ cells. There are at least three major subtypes of CAFs, namely, myofibroblastic (myCAF), inflammatory (iCAF), and antigen-presenting (apCAF) (*58*, *69*, *70*). Notably, MHC-II is highly expressed on all apCAFs and some iCAFs. It has been proposed that MHC-II is involved in promoting the dysfunction of conventional CD4^+^ T cells in the TME (*58*). It has also been shown that MHC-II expressed on lymph node stromal cells promotes T_reg_ cells (*71*, *72*). Therefore, it is possible that CAF-expressed self-antigen peptide–MHC-II complexes engage and stimulate T_reg_ cells in the TME. In keeping with this notion, a recent report demonstrates that MHC-II expressed in CAFs promotes the generation of induced T_reg_ cells in vitro (*73*). In addition, CAFs have been found to produce IL-33, which promotes tumor metastasis and immunosuppression through T_reg_ cells (*27*, *29*, *74*). Our results show a correlation between the expression of the genes in the MHC-II antigen presentation pathway in CAFs and the number and clonal expansion of IL1RL1^+^ T_reg_ cells. This is consistent with the idea that CAFs directly stimulate and promote the accumulation of T_reg_ cells in the TME. Our data further indicates that T_reg_-expressed AREG is required for the expression of MHC-II in CAFs and clonal expansion of T_reg_ cells. These findings support the notion that CAF-expressed MHC-II promotes the accumulation of T_reg_ cells in the TME, thereby driving tumor immune tolerance.

Our results suggest a hypothesis that CAFs have complex roles in tumor progression and antitumor immune responses due to two major functional states, i.e., tissue repairing and immune-boosting states. The fibroblasts in the tissue-repairing state promote tissue repair and inhibit immune responses, whereas the fibroblasts in the immune-boosting state mediate inflammation and immune responses. This study demonstrates that the AREG/EGFR axis enables cross-talk between IL1RL1^+^ T_reg_ cells and CAFs and plays an important role in driving the tissue repairing and immunosuppressive state of CAFs. This functional state is crucial for promoting tumorigenesis and maintaining cancer immune tolerance.

## MATERIALS AND METHODS

### Mouse

C57BL/6J (stock number 000664) and Foxp3^YFP-cre^ (stock number 016959) mice were purchased from the Jackson Laboratory. The ST2 flox (strain ID: Il1rl1^tm1a(KOMP)^) mice were purchased from KOMP Repository (UC Davis). All mice are on the background of C57BL/6. Mice were housed in the specific pathogen–free facility in the School of Medicine, University of Pittsburgh. All mice experiments were performed under the approval of the Institutional Animal Care and Use Committee of the University of Pittsburgh.

### Cell lines and animal models

MC38 cell line was generously provided by Z. Guo (University of Pittsburgh, School of Medicine) and cultured in Dulbecco’s modified Eagle’s medium with 10% fetal bovine serum (FBS) and 1% penicillin-streptomycin (P.S). Generation of B16–IL-33 and B16-vec tumor cell lines was previously reported (*10*). All cell lines were tested for mycoplasma routinely and with negative results. B16 and B16–IL-33 cells were cultured in RPMI 1640 medium with 10% FBS and 1% P.S. One million MC38 cells were injected intradermally into the right flank of the mice. For B16 models, 0.1 million cells were injected intradermally. IL-33 protein (10 μg), anti–PD-1 (200 μg per mouse; Bio X Cell, clone: J43), or anti-Areg (50 μg per mouse; R&D Systems, clone: 206220) was injected intraperitoneally on the fifth day after tumor inoculation, for a total of four times with 4-day intervals.

### Isolation of single cells from tumor tissues

Tumor tissues were processed according to the protocol described previously (*75*). Briefly, mice were euthanized, and tissues were freshly dissected. Tumor tissues were then cut into pieces and digested in serum-free RPMI 1640 with deoxyribonuclease (0.33 mg/ml; Sigma-Aldrich) and Liberase TL (0.25 mg/ml; Roche) and then ground, washed in PBS, and filtered through a 70-μm cell strainer for single-cell suspensions. CD45 (TIL) microbeads (Miltenyi Biotec) were used for the isolation of tumor-infiltrating leukocytes from mouse tumors following the manufacturer’s protocol.

### Flow cytometry

Flow cytometry experiments were performed on Aurora (Cytek Biosciences), BD Symphony 3 Flow cytometer, BD FACS ARIA III Sorter, and BD Melody Cell Sorter from the flow core in the University of Pittsburgh or Center for Discovery and Innovation and analyzed by Flowjo (BD). CD45 (clone 30-F11), CD4 (clone RMT4-5), CD8a (clone 53.67), Tcf1/Tcf7 (clone C63D9), PD-1 (clone J43), Tim-3 (clone RMT3-23), Lag-3 (clone C9B7W), Thy1.2 (clone 53-2.1), IFN-γ (clone XMG1.2), Ly-6C (clone HK1.4), Ly-6G (clone 1A8), Sca-1 (clone D7), ST2 (clone RMST2-33), CD103 (clone M290), GzmB (clone QA16A02), Foxp3 (clone MF-14), Ki-67 (clone 16A8), CD11b (clone M1/70), CD11c (clone N418), CD86 (clone GL-1), MHCII (clone), F4/80 (clone BM8), CD206 (clone C068C2), Arg1 (clone A1exF5), CD25 (clone PC61), CD62L (clone MEL-14), CD44 (clone IM7), CD90.2 (clone 53-2.1), CD140a (clone APA5), LIVE/DEAD dye (Zombie NIR Dye) were purchased from BD Bioscience, Thermo Fisher Scientific, or BioLegend. For intracellular transcription factors and cytokines staining, cells were stimulated with a leukocyte activation cocktail (BD) for 6 hours and then followed the standard staining protocol described previously (*49*).

### DNA extraction and PCR

All the samples were processed with Extracta DNA prep for polymerase chain reaction (PCR) (Quantabio), following the manufacturer’s instructions. PCR was carried out through MJ Mini Thermal Cycler (Bio-Rad) using the OneTaq Quick-Load 2X Master Mix with standard buffer (New England Biolabs). Primer sequences are provided in table S1.

### RNA extraction and nested PCR

Total RNA was isolated using the RNeasy Mini Kit according to the manufacturer’s instructions (QIAGEN). Synthesis of cDNA was performed by using the qScript cDNA Synthesis Kit (Quantabio). First amplification reaction was carried out through MJ Mini Thermal Cycler (Bio-Rad) using the OneTaq Quick-Load 2X Master Mix with standard buffer (New England Biolabs). The PCR amplification product was used as the template for the nested PCR conducted with QuantStudio 5 (Applied Biosystems) using the PowerUp SYBR Green Master Mix (Thermo Fisher Scientific). The primer sequences are listed in table S1. Glyceraldehyde-3-phosphate dehydrogenase expression served as an internal standard. Relative gene expression was determined by using the 2^−ΔΔCT^ comparative method.

### Droplet-based scRNA-seq

CD4^+^ and CD8^+^ T cells were purified by fluorescence-activated cell sorting and washed twice in PBS with bovine serum albumin (BSA; 400 μg/ml) and then resuspended with the concentration of 1000 to 2000 cells/μl. A total of 18,000 cells were loaded into the Chromium single-cell A chip to generate Gel Bead-In Emulsions using the 10x Genomics 5′ RNA single-cell method. Chromium Single Cell 5′ and Chromium Single Cell V(D)J Reagent Kits (10x Genomic, no. CG000086) were used to generate single-cell sequencing libraries. Libraries were sequenced on an Illumina NovaSeq6000 SP platform with 40,000 reads per cell on average.

### CITE-seq

We used Cellular Indexing of Transcriptomes and Epitopes by Sequencing (CITE-seq) and Cell Hashing antibodies to achieve a single-cell level protein expression data and demultiplexing of different samples. Briefly, single-cell suspensions from tumor tissues were washed with staining buffer (PBS, 2% BSA, and 0.01% Tween) and incubated with Fc blocker (BioLegend, Trustain FcX) for 10 min on ice. Antibody cocktails were prepared by mixing CITE-seq antibodies, cell hashing tags, and fluorescent-labeled antibodies with preoptimized concentrations. Antibody cocktails were applied, and cells were incubated on ice for 30 min. Stained cells were washed three times using staining buffer, then CD4^+^ and CD8^+^ T cells from different treatment groups were sorted out and pooled immediately before loading to a 10x single-cell platform. We used 10x Chromium Single Cell 5′ V(D)J Reagent Kits (10x Genomics, no. CG000186) to construct the cell surface protein libraries. Libraries were sequenced on an Illumina NovaSeq6000 SP platform with 10,000 reads per cell on average.

### scRNA-seq data processing

The sequenced raw read data were aligned to mouse reference genome mm10 and collapsed by barcode and unique molecular identifier (UMI) by Cell Ranger (10x). The Seurat (3.1.5) R package was used to identify clusters and find differentially expressed genes. The Seurat object was set up by filtering out genes expressed on less than four cells and cells with less than 200 detected features. Cells with unique feature counts between 200 and 2500 and less than 5% mitochondrial counts were selected for further analysis. Following the standard quality control workflow, the UMI count matrix was log-transformed, normalized, and scaled using default parameters.

### scRNA-seq dataset dimension reduction

For Seurat version 3.1.5 functions, FindVariableFeatures, RunPCA, and RunUMAP were used to calculate top variable genes, principal components analysis (PCA), and UMAP, respectively. We used the top 2000 features to calculate PCA and used the top 50 principal components (PCs) to generate UMAP visualizations. Shared nearest neighbor clustering method was performed by Seurat’s FindClusters function based on the top 50 PCs, with resolution set to 0.8 to 1.4.

### Identify GEMs using the nHDP model

We applied the nHDP model on mouse scRNA-seq data to identify the GEMs that potentially indicate different biological processes (*76*). The nHDP model is originally designed to model co-occurrence patterns of words in text documents in the text mining domain, which is a close analog to coexpression expression of genes (words) in single cells (documents). Furthermore, it hierarchically organizes GEMs in a tree, so that the GEMs close to the root are expressed in a broad range of cells, whereas the GEMs at the leaves of the tree are only expressed in highly specialized (differentiated) cells. We designed a three-layer hierarchical tree structure, with branching factors of 5, 4, and 3 from the root to the second and third layers leading to 85 nodes per GEMs in total. Each GEM defines a distribution over the space of genes reflecting the information on which genes are commonly assigned to the module. The nHDP model generates (i) a ranked list of genes most commonly assigned to GEMs and (ii) a cell-by-GEM count matrix, of which an element reflects the number of genes expressed in a given cell that are assigned to a specific GEM.

### Test differentially expression of a GEM between subpopulations of single cells

We use the nonparametric Wilcoxon rank sum test to test whether a GEM is differentially expressed between know-out cells and wild-type cells (*77*). We set detection threshold (α), at 0.05, when assessing whether the GEM is significantly differentially expressed.

### Analysis of transcription factors in scRNA-seq data

The SCENIC pipeline (R package, v.1.1.2.2) was used to construct and score gene regulatory networks (regulons) as described previously (*53*). Each regulon is composed of a transcription factor and its putative target genes. Next, the regulon activities of each cell were scored by AUCell. The output of SCENIC is a matrix of the activity of regulons, where rows correspond to regulons and columns correspond to cells. Transcription factor binding motif analysis of putative genes in the regulon was performed with HOMER (v4.10) “findMotifs.pl” (-start -500 -end 500). Differentially expressed regulons in each cluster or in each condition were calculated by Wilcoxon test.

### Visualization of ATAC-seq data

To examine the Areg expression in ST2 T_reg_ cells and other T_reg_ cells, we analyzed publicly available ATAC-seq datasets (GSE130884) of sorted tissue ST2 T_reg_ cells and other T_reg_ cells from lung tissue (*78*). ATAC-seq tracks were visualized in the Integrative Genomics Viewer (V2.7.2).

### Statistical analysis

Statistical analysis was performed using Graphpad Prism v8 software. Values were reported as means ± SEM. *P* value was calculated by two-tailed Student’s *t* test when comparing two groups and the log-rank (Mantel-Cox) test for comparing several Kaplan-Meier survival curves. Two-way analysis of variance (ANOVA) was used for comparing tumor growth curves. Exact one-proportion *z* test was used for comparing the proportions of different samples. *P < 0.05, **P < 0.01, ***P < 0.001, and ****P < 0.0001.
