# Supplementary material for: Amphiregulin couples IL1RL1+ regulatory T cells and cancer-associated fibroblasts to impede antitumor immunity
Source: Sci Adv. 2023 Aug 23;9(34):eadd7399. doi: 10.1126/sciadv.add7399 (PMC10446484; doi:10.1126/sciadv.add7399)
Supplement: Supplementary file 1 — Figs. S1 to S8 [file sciadv.add7399_sm.pdf]

Science Advances
AAAS

Supplementary Materials for

Amphiregulin couples IL1RL1^{+} regulatory T cells and cancer-associated fibroblasts to impede antitumor immunity

Runzi Sun *et al.*

Corresponding author: Binfeng Lu, binfeng.lu@hmh-cdi.org

Sci. Adv. **9**, eadd7399 (2023)
DOI: 10.1126/sciadv.add7399

This PDF file includes:

Figs. S1 to S8

figure S1

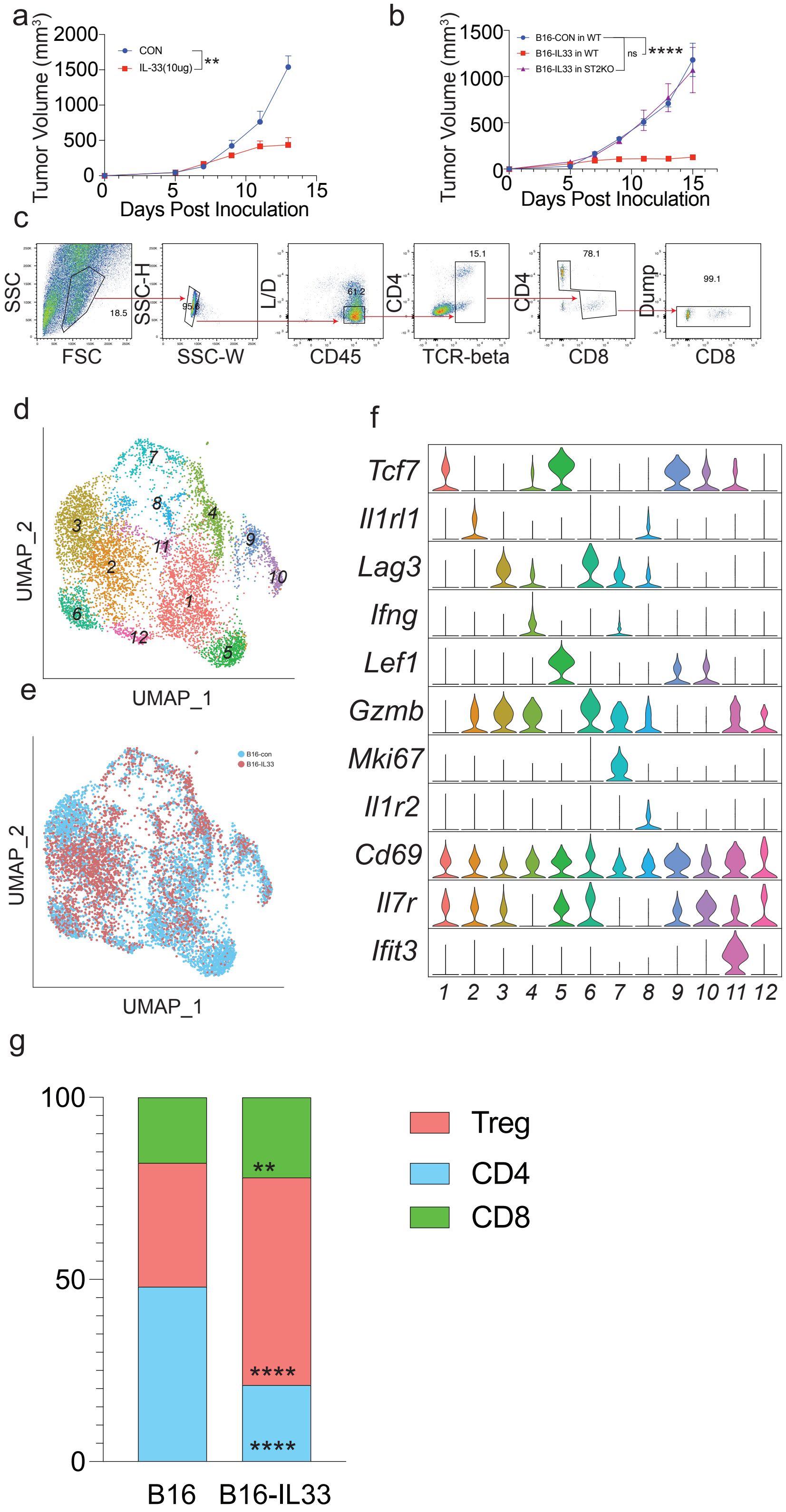

a
Tumor Volume (mm3)
2000
1500
1000
500
0
CON
IL-33(10ug)
**
Days Post Inoculation
b
1500
1000
500
B16-CON in WT
B16-IL33 in WT
B16-IL33 in ST2KO
ns
****
c
SSC
FSC
18.5
SSC-H
SSC-W
L/D
CD45
61.2
CD4
TCR-beta
15.1
78.1
CD8
Dump
99.1
d
UMAP_2
UMAP_1
e
B16-con
B16-IL33
f
Tcf7
Il1rl1
Lag3
Ifng
Lef1
Gzmb
Mki67
Il1r2
Cd69
Il7r
Ifit3
1 2 3 4 5 6 7 8 9 10 11 12
g
100
50
0
B16
B16-IL33
Treg
CD4
CD8
**
****
****

Figure S1.

scRNAseq analysis of the TME of B16 and B16-IL33.

(a) B16 tumor cells ($1x10^5$) were inoculated i.d. into the right flank of the C57BL/6J mice, IL-33 protein and PBS was treated starting from day 5 and again every 4 days for a total of 3 times. Tumor sizes were monitored every 2 days, average sizes were shown. (b) B16 or B16-IL33 tumor cells ($1x10^5$) were inoculated *i.d.* into the right flank of the C57BL/6J mice or ST2 knock out mice. Tumor sizes were monitored every 2 days, average sizes were shown. (c) Gating strategy to cover T cells from B16 and B16-IL33 tumors for single cell RNA sequencing. (d) UMAP dimensionality reduction projects day 9 T cells from B16 and B16-IL33 tumors to 2 dimensions showing 12 subclusters differentiated by color. Each point represents a single cell, with cells of similar gene expression profiles positioned closer together in the projection. (e) UMAP projection comparing the distribution of T cells in B16 and B16-IL33 tumors.

(f) Unsupervised clustering identifies 12 clusters based on expression profiles. Violin plot shows the expression of the top marker gene for all 12 clusters based on adjusted P-value and log2FC.

(g) The bar plot shows the percent distribution of these cell types in B16 T cells compared with B16-IL33 T cells. The data shown are representative of three independent experiments. Graphs shown represented data summarized as means ± SEM and were analyzed by two-way analysis of variance (ANOVA), $^{**}P < 0.01$, and $^{****}P < 0.0001$.

figure S2

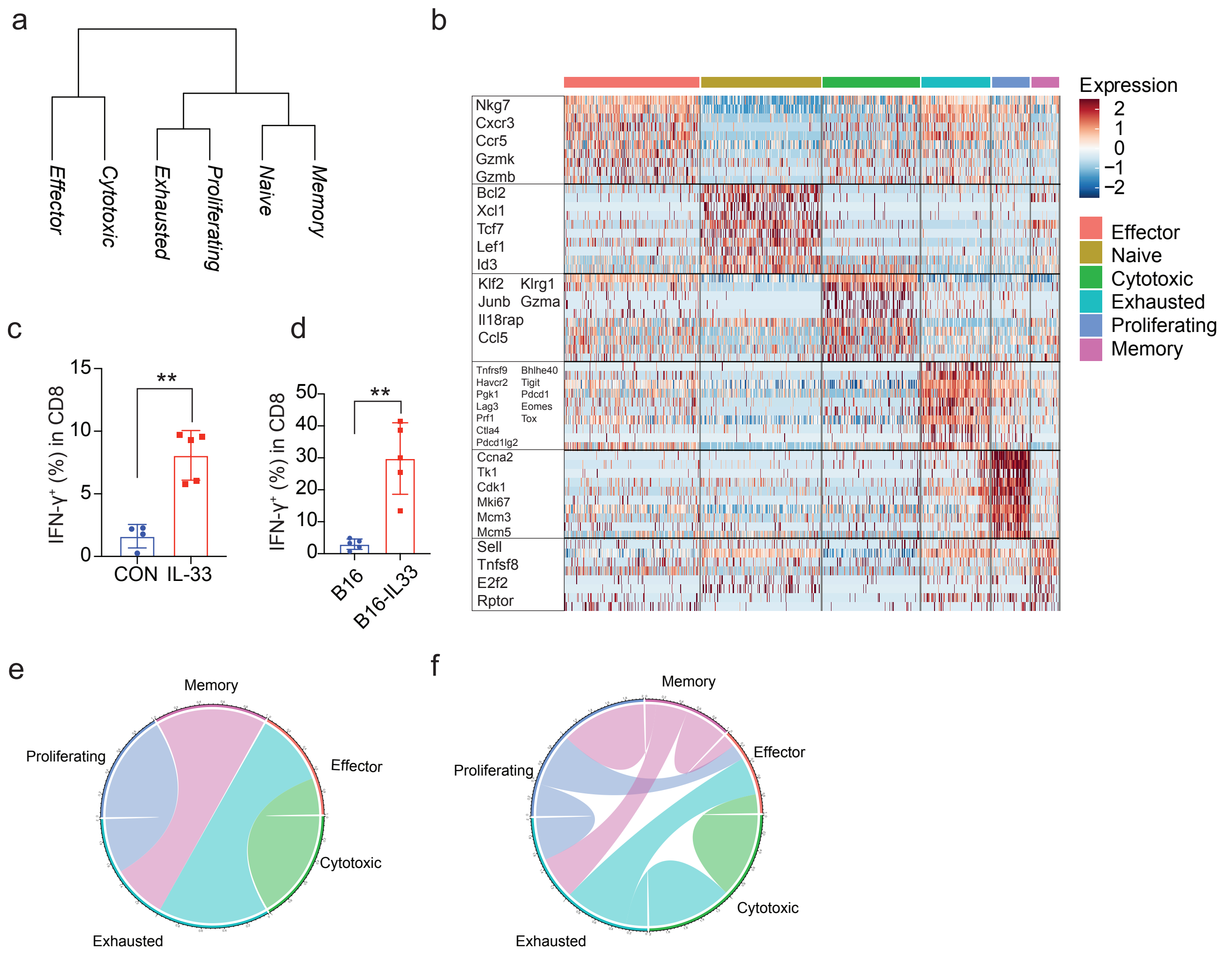

a
Effector
Cytotoxic
Exhausted
Proliferating
Naive
Memory
b
Expression
2
1
0
−1
−2
Effector
Naive
Cytotoxic
Exhausted
Proliferating
Memory
Nkg7
Cxcr3
Ccr5
Gzmk
Gzmb
Bcl2
Xcl1
Tcf7
Lef1
Id3
Klf2 Klrg1
Junb Gzma
Il18rap
Ccl5
Tnfrsf9 Bhlhe40
Havcr2 Tigit
Pgk1 Pdcd1
Lag3 Eomes
Prf1 Tox
Ctla4
Pdcd1lg2
Ccna2
Tk1
Cdk1
Mki67
Mcm3
Mcm5
Sell
Tnfsf8
E2f2
Rptor
c
IFN-γ⁺ (%) in CD8
15
10
5
0
**
CON
IL-33
d
IFN-γ⁺ (%) in CD8
50
40
30
20
10
0
**
B16
B16-IL33
e
Memory
Proliferating
Effector
Cytotoxic
Exhausted
f
Memory
Effector
Proliferating
Cytotoxic
Exhausted

Figure S2.

scRNAseq and flow cytometric analysis of $CD8^+$ T cells in the TME of B16 and B16-IL33.

(a) Hierarchical clustering of all six $CD8^+$ T cell clusters based on average gene expression. (b) Heatmap of differentially expressed genes of each $CD8^+$ T cell cluster. (c) Bar plot showing the percentage of IFN-γ^+ CD8 T cells in PBS or IL-33 treated MC38 tumor bearing mice. (d) Bar plot showing the percentage of IFN-γ^+ CD8 T cells in B16 and B16-IL-33 tumor bearing mice. (e-f) Chord plot showing the detailed TCR sharing situations across six $CD8^+$ T cell clusters in B16 (e) and B16-IL33 (f). The data shown are representative of three independent experiments. Graphs shown represented data summarized as means ± SEM and were analyzed by unpaired two-tailed Student’s t-test, $^{**}P < 0.01$.

figure S3

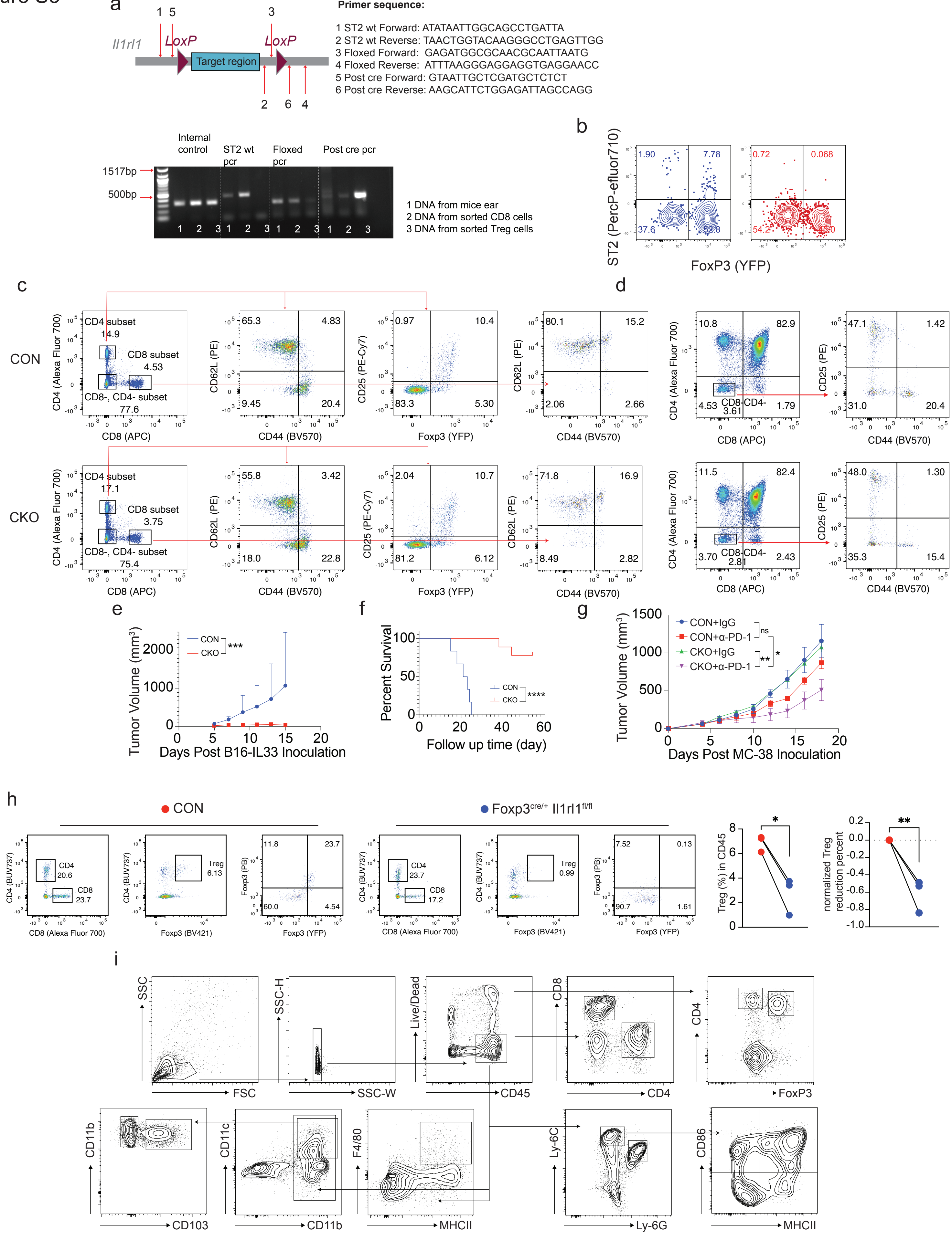
a
Il1rl1
LoxP
Target region
LoxP
Primer sequence:
1 ST2 wt Forward: ATATAATTGGCAGCCTGATTA
2 ST2 wt Reverse: TAACTGGTACAAGGGCCTGAGTTGG
3 Floxed Forward: GAGATGGCGCAACGCAATTAATG
4 Floxed Reverse: ATTTAAGGGAGGAGGTGAGGAACC
5 Post cre Forward: GTAATTGCTCGATGCTCTCT
6 Post cre Reverse: AAGCATTCTGGAGATTAGCCAGG
Internal control
ST2 wt pcr
Floxed pcr
Post cre pcr
1517bp
500bp
1 DNA from mice ear
2 DNA from sorted CD8 cells
3 DNA from sorted Treg cells
b
ST2 (PercP-efluor710)
FoxP3 (YFP)
c
CON
CKO
CD4 subset
CD8 subset
CD8-, CD4- subset
CD4 (Alexa Fluor 700)
CD8 (APC)
CD62L (PE)
CD44 (BV570)
CD25 (PE-Cy7)
Foxp3 (YFP)
d
CD25 (PE)
e
Tumor Volume (mm³)
Days Post B16-IL33 Inoculation
CON
CKO
***
f
Percent Survival
Follow up time (day)
****
g
CON+IgG
CON+α-PD-1
CKO+IgG
CKO+α-PD-1
ns
**
*
Days Post MC-38 Inoculation
h
CON
Foxp3cre/+ Il1rl1fl/fl
CD4 (BUV737)
CD8 (Alexa Fluor 700)
Foxp3 (BV421)
Foxp3 (PB)
Foxp3 (YFP)
Treg
Treg (%) in CD45
normalized Treg reduction percent
i
SSC
FSC
SSC-H
SSC-W
Live/Dead
CD45
CD8
CD4
FoxP3
CD11b
CD103
CD11c
F4/80
MHCII
Ly-6C
Ly-6G
CD86

Figure S3.

Deletion of Il1rl1 in Treg cells resulted in stronger antitumor efficacy in both IL-33-treated and PD-1 mAbs treated mouse tumor models.

(a) Schematic diagram indicating the location of primers. Treg cells and CD8 T cells were purified by FACS from Foxp3Cre Il1rl1 fl/fl mice. Then the DNA was extracted along with tail specimens. PCR amplified bands were shown. (b) representative flow cytometry plot showing the ST2 staining in Treg cells. (c-d) Cytometry gating of the spleen (c) and thymus (d) for T cell populations in CON and CKO mice. (e-f). B16-IL33 tumor cells (1x105) were inoculated i.d. into the right flank of the male CON(Foxp3^{cre}) and CKO (Foxp3^{cre}Il1rl1$^{flox/flox}$) mice. Tumor size was monitored every two days. Tumor curve (e) and overall survival (f) of B16-IL33 tumor-bearing mice. (g) Tumor curve of MC38 tumor-bearing CON or CKO mice treated with anti-PD-1 antibody. (h) Representative flow cytometry plots and statistical quantifications of Treg cells and Foxp3 or YFP expression in CD4 T cells from three B16-IL33 tumors in control and foxp3$^{cre/+}$ Il1rl1$^{fl/fl}$ mice. (i) Flow cytometry gating strategy. Tumor-infiltrating lymphocytes and myeloid cells analysis for Figure 3 and Figure 4. The data shown are representative of two independent experiments. Graphs shown represented data summarized as means ± SEM and were analyzed by unpaired two-tailed Student’s t-test or two-way analysis of variance (ANOVA), $^{*}P < 0.05$, $^{**}P < 0.01$, $^{***}P < 0.001$, and $^{****}P < 0.0001$.

figure S4

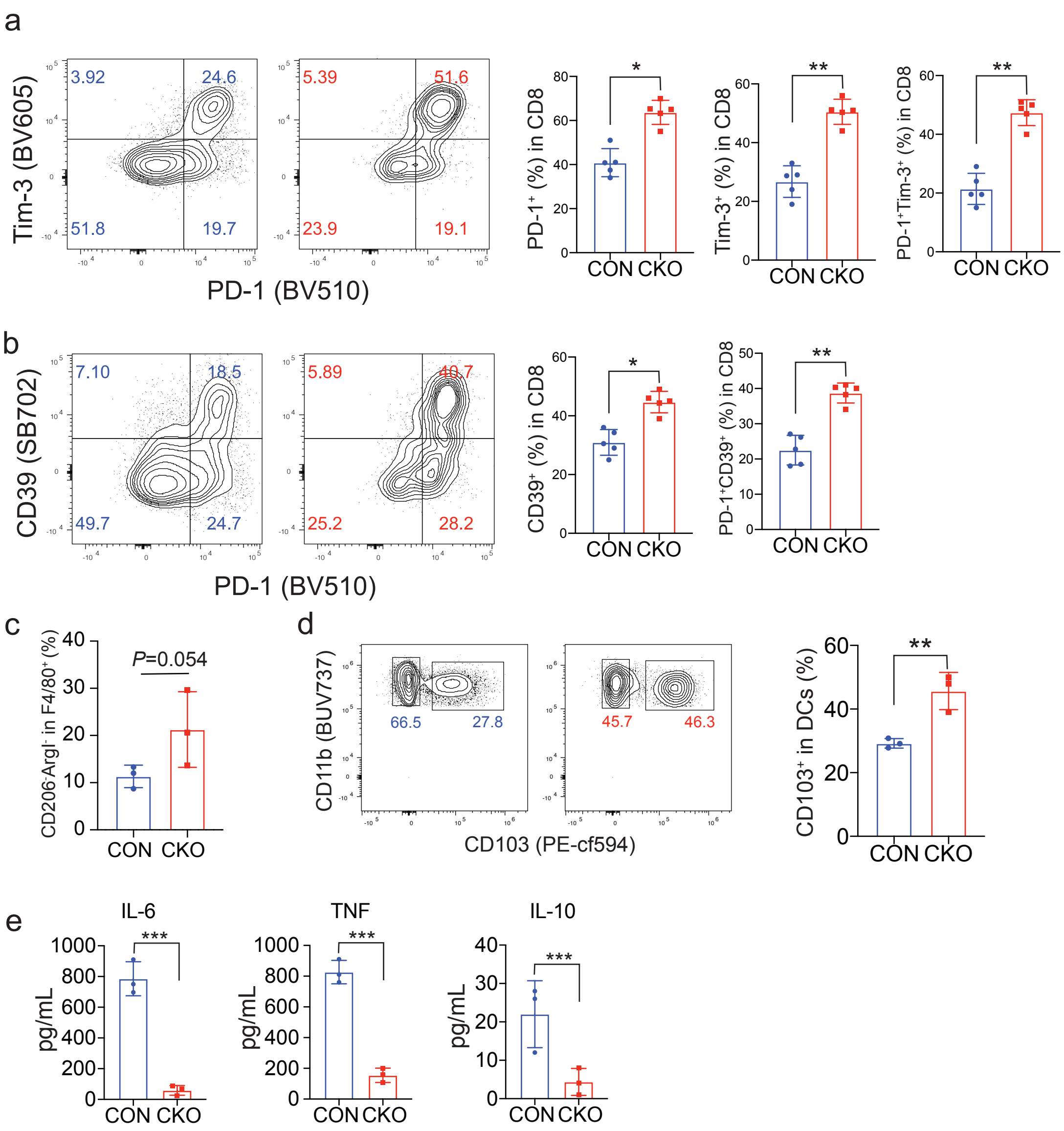

a
Tim-3 (BV605)
PD-1 (BV510)
3.92
24.6
51.8
19.7
5.39
51.6
23.9
19.1
PD-1+ (%) in CD8
Tim-3+ (%) in CD8
PD-1+Tim-3+ (%) in CD8
CON CKO
b
CD39 (SB702)
PD-1 (BV510)
7.10
18.5
49.7
24.7
5.89
40.7
25.2
28.2
CD39+ (%) in CD8
PD-1+CD39+ (%) in CD8
c
CD206-ArgI- in F4/80+ (%)
P=0.054
d
CD11b (BUV737)
CD103 (PE-cf594)
66.5
27.8
45.7
46.3
CD103+ in DCs (%)
e
IL-6
TNF
IL-10
pg/mL

Figure S4.

The changes of T cells and DCs in the TME upon deletion of Il1rl1 in Treg cells.

(a) Representative flow cytometry plot and quantitative plot of the percentage of PD-1$^+$, Tim-3$^+$, or PD-1$^+$Tim-3$^+$ CD8$^+$ T cells. (b) Representative flow cytometry plot and quantitative plot of the percentage of 39$^+$ or PD-1$^+$CD39$^+$ CD8$^+$ T cells. (c) Bar plot showing the percentage of type1 macrophages in CON(*Foxp3*^{cre}) or CKO (*Foxp3*^{cre}*Il1rl1*$^{flox/flox}$) mice. (d) Representative flow cytometry plot and a quantitative plot showing the percentage of CD103$^+$ dendritic cells. (e) Bar plot showing the IL-6, TNF, and IL-10 levels in tumor extracts from CON or CKO mice. Data shown represent two to five independent experiments (day8 takedown). Graphs shown represented data summarized as means ± SEM and were analyzed by unpaired two-tailed Student's t-test, *$P < 0.05$, **$P < 0.01$, and ***$P < 0.001$.

figure S5

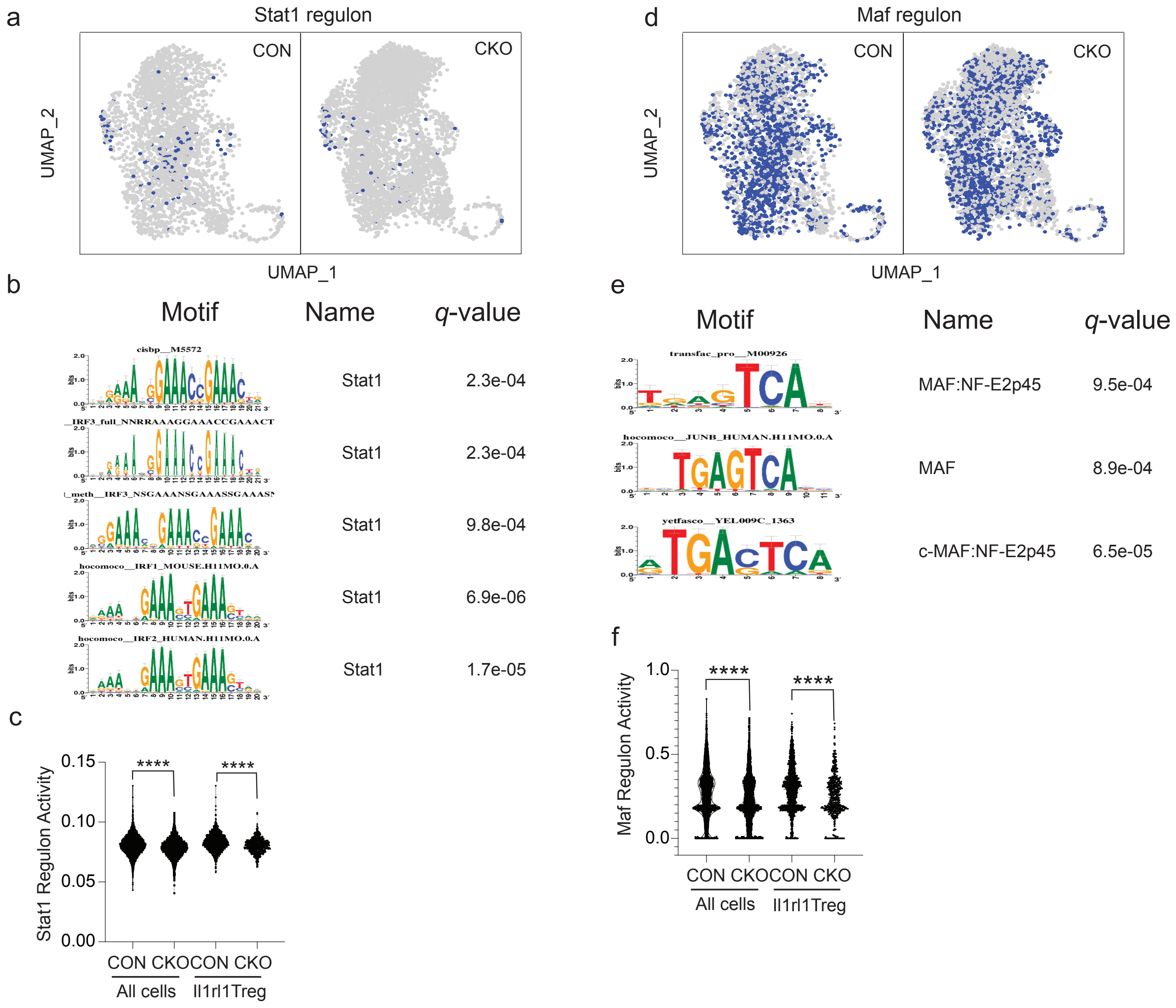
a
Stat1 regulon
CON
CKO
UMAP_2
UMAP_1
b
Motif
Name
q-value
Stat1
2.3e-04
Stat1
2.3e-04
Stat1
9.8e-04
Stat1
6.9e-06
Stat1
1.7e-05
c
Stat1 Regulon Activity
0.15
0.10
0.05
0.00
****
****
CON CKO CON CKO
All cells
Il1rl1Treg
d
Maf regulon
CON
CKO
UMAP_2
UMAP_1
e
Motif
Name
q-value
MAF:NF-E2p45
9.5e-04
MAF
8.9e-04
c-MAF:NF-E2p45
6.5e-05
f
Maf Regulon Activity
1.0
0.5
0.0
****
****
CON CKO CON CKO
All cells
Il1rl1Treg

Figure S5.

IL-33-induced transcription factors in IL1RL1^{+} Treg cells.

(a) UMAP projection comparing the distribution of Stat1 regulon in B16 and B16-IL33 tumors. (b) Motif enriched in the promoter region of Stat1 target genes. (c) Violin plot showing the Stat1 regulon activity of Treg cells in all clusters and in ST2Treg cluster. (d) UMAP projection comparing the distribution of Maf regulon in B16 and B16-IL33 tumors. (e) Motif enriched in the promoter region of Maf target genes. (f) Violin plot showing the Maf regulon activity of Treg cells in all clusters and the ST2^{+} Treg cluster. ****P < 0.0001.

figure S6

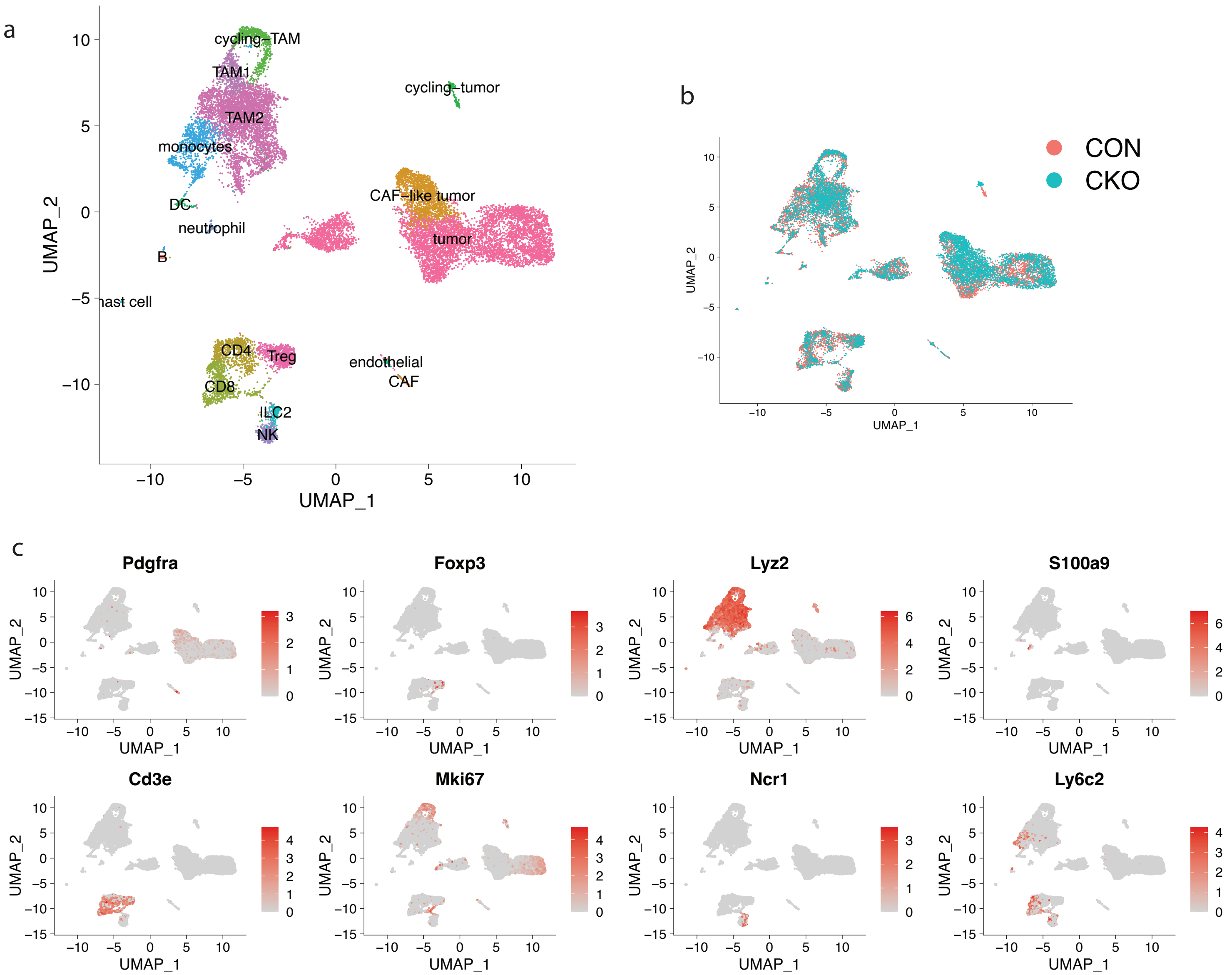
a
cycling−TAM
TAM1
TAM2
monocytes
DC
neutrophil
B
mast cell
cycling−tumor
CAF−like tumor
tumor
CD4
Treg
CD8
ILC2
NK
endothelial
CAF
UMAP_1
UMAP_2
b
CON
CKO
UMAP_1
UMAP_2
c
Pdgfra
Foxp3
Lyz2
S100a9
Cd3e
Mki67
Ncr1
Ly6c2
UMAP_1
UMAP_2

Figure S6.

The whole tumor scRNAseq analysis of MC38 tumors upon IL-33 treatment.

(a) UMAP dimensionality reduction projects all cells from PBS or IL-33 treated MC38 tumor to 2 dimensions showing different subclusters differentiated by color. Each point represents a single cell, with cells of similar gene expression profiles positioned closer together in the projection. (b) UMAP projection comparing the distribution of cells from PBS or IL-33 treated MC38. (c) UMAP projection of several cluster-specific genes.

figure S7

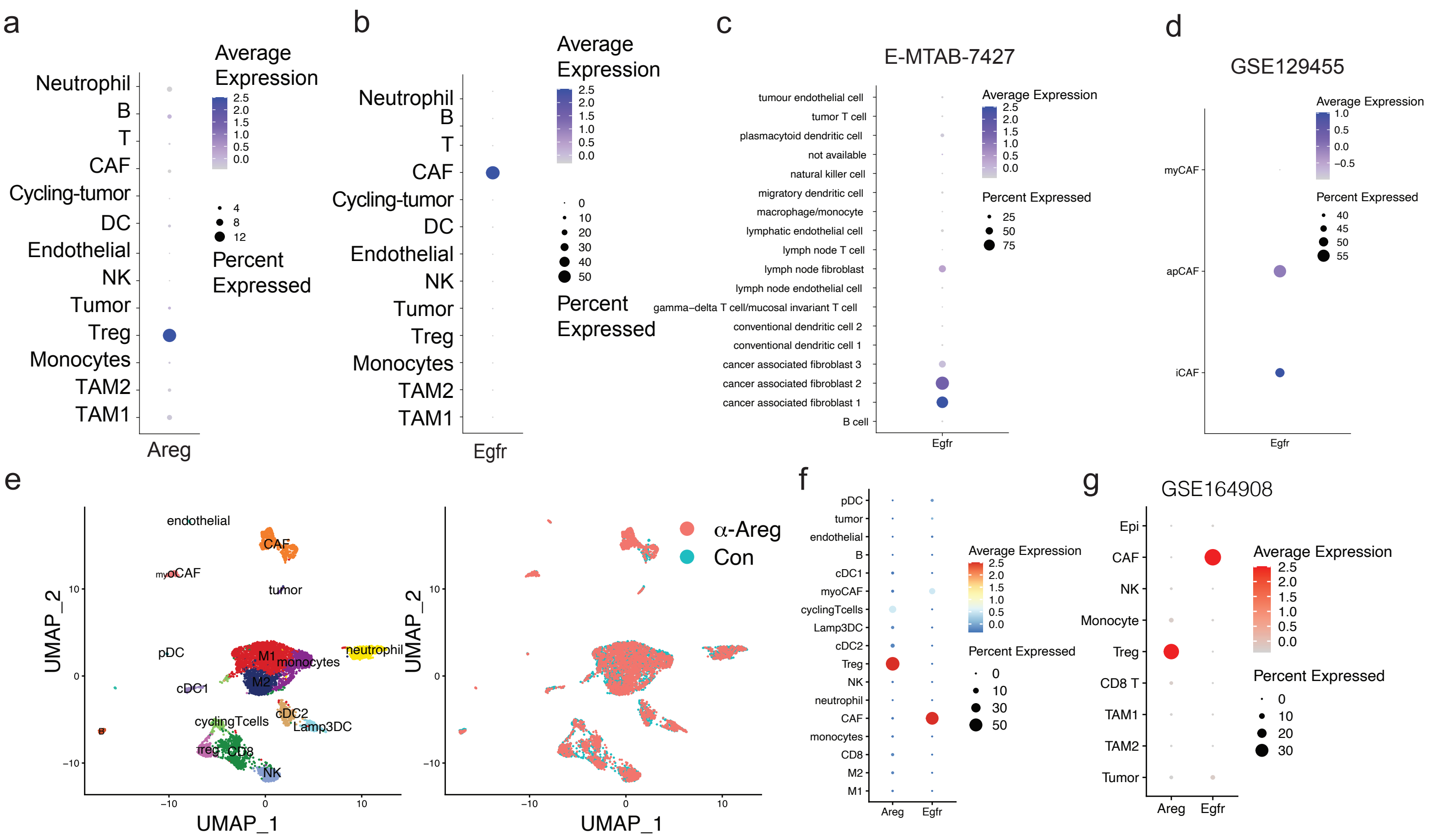
a
Average Expression
Percent Expressed
Neutrophil
B
T
CAF
Cycling-tumor
DC
Endothelial
NK
Tumor
Treg
Monocytes
TAM2
TAM1
Areg
b
Egfr
c
E-MTAB-7427
tumour endothelial cell
tumor T cell
plasmacytoid dendritic cell
not available
natural killer cell
migratory dendritic cell
macrophage/monocyte
lymphatic endothelial cell
lymph node T cell
lymph node fibroblast
lymph node endothelial cell
gamma–delta T cell/mucosal invariant T cell
conventional dendritic cell 2
conventional dendritic cell 1
cancer associated fibroblast 3
cancer associated fibroblast 2
cancer associated fibroblast 1
B cell
d
GSE129455
myCAF
apCAF
iCAF
e
UMAP_1
UMAP_2
endothelial
CAF
myoCAF
tumor
pDC
M1
monocytes
neutrophil
M2
cDC1
cDC2
cyclingTcells
Lamp3DC
Treg
CD8
NK
α-Areg
Con
f
pDC
tumor
endothelial
B
cDC1
myoCAF
cyclingTcells
Lamp3DC
cDC2
Treg
NK
neutrophil
CAF
monocytes
CD8
M2
M1
g
GSE164908
Epi
CAF
NK
Monocyte
Treg
CD8 T
TAM1
TAM2
Tumor

Figure S7.

***Areg* and *Egfr* expression in tumor samples.**

(a) The dot plot shows the *Areg* gene expression level across all clusters in the MC38 tumor microenvironment related in figure S6. (b) Dot plot showing the *Egfr* gene expression level across all clusters in the MC38 tumor microenvironment. (c) The dot plot shows the *Egfr* gene expression level across all clusters in the B16 tumor microenvironment. Data reanalyzed from E-MTAB-7427 (d) Dot plot showing the *Egfr* gene expression level across all CAF clusters in the PDAC cancer microenvironment. Data reanalyzed from GSE129455. (e) UMAPs show cells within the B16-IL33 tumors from control and anti-Areg treated mice. (f) The dot plot shows the expression of Areg and Egfr in each cell type in B16-IL33 tumors. (g) The dot plot shows the expression of Areg and Egfr across different cell types in the TME of MC38 tumors. Data from GSE164908 were reanalyzed.

figure S8

a

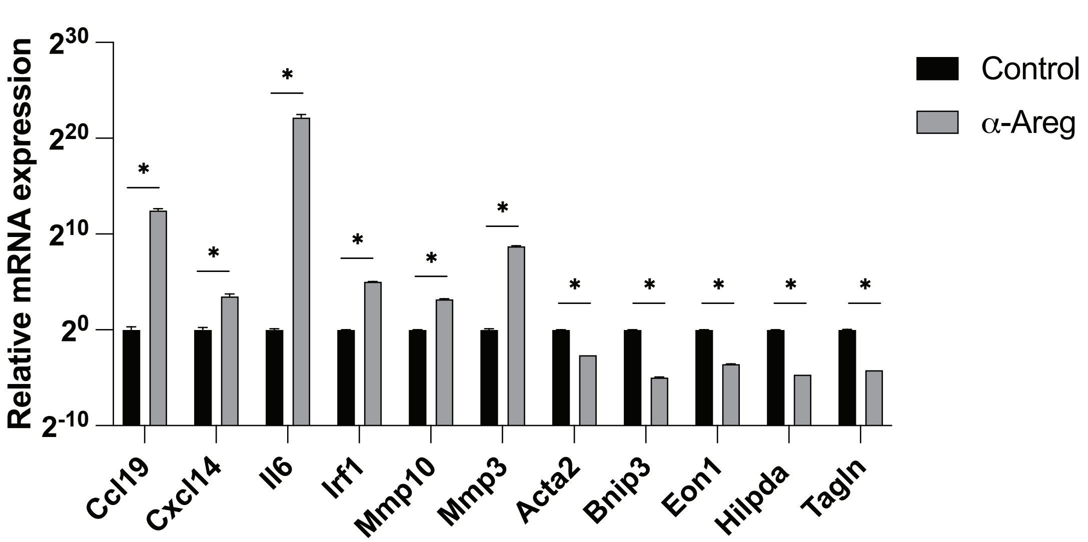

Relative mRNA expression
2^30
2^20
2^10
2^0
2^-10
*
Control
α-Areg
Ccl19
Cxcl14
Il6
Irf1
Mmp10
Mmp3
Acta2
Bnip3
Eon1
Hilpda
Tagln

b

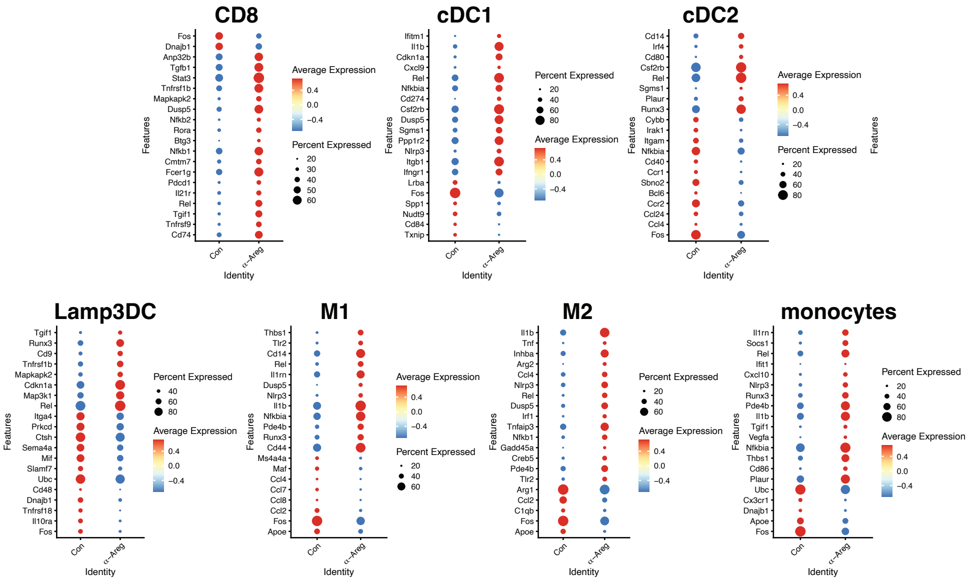

CD8
Fos
Dnajb1
Anp32b
Tgfb1
Stat3
Tnfrsf1b
Mapkapk2
Dusp5
Nfkb2
Rora
Btg3
Nfkb1
Cmtm7
Fcer1g
Pdcd1
Il21r
Rel
Tgif1
Tnfrsf9
Cd74
Average Expression
Percent Expressed
cDC1
Ifitm1
Il1b
Cdkn1a
Cxcl9
Rel
Nfkbia
Cd274
Csf2rb
Dusp5
Sgms1
Ppp1r2
Nlrp3
Itgb1
Ifngr1
Lrba
Fos
Spp1
Nudt9
Cd84
Txnip
cDC2
Cd14
Irf4
Cd80
Csf2rb
Rel
Sgms1
Plaur
Runx3
Cybb
Irak1
Itgam
Nfkbia
Cd40
Ccr1
Sbno2
Bcl6
Ccr2
Ccl24
Ccl4
Fos
Lamp3DC
Tgif1
Runx3
Cd9
Tnfrsf1b
Mapkapk2
Cdkn1a
Map3k1
Rel
Itga4
Prkcd
Ctsh
Sema4a
Mif
Slamf7
Ubc
Cd48
Dnajb1
Tnfrsf18
Il10ra
Fos
M1
Thbs1
Tlr2
Cd14
Rel
Il1rn
Dusp5
Nlrp3
Il1b
Nfkbia
Pde4b
Runx3
Cd44
Ms4a4a
Maf
Ccl4
Ccl7
Ccl8
Ccl2
Fos
Apoe
M2
Il1b
Tnf
Inhba
Arg2
Ccl4
Nlrp3
Rel
Dusp5
Irf1
Tnfaip3
Nfkb1
Gadd45a
Creb5
Pde4b
Tlr2
Arg1
Ccl2
C1qb
Fos
Apoe
monocytes
Il1rn
Socs1
Rel
Ifit1
Cxcl10
Nlrp3
Runx3
Pde4b
Il1b
Tgif1
Vegfa
Nfkbia
Thbs1
Cd86
Plaur
Ubc
Cx3cr1
Dnajb1
Apoe
Fos
Features
Identity
Con
α-Areg

Figure S8.

Anti-Areg antibody altered tumor microenvironment.

(a) A bar plot shows RT-QPCR results of the top differentially expressed genes in CAFs purified from control and the anti-Areg-antibody-treated mice. This panel is related to Figures 6f and 6g.

(b) Dot plots of the scRNAseq analysis illustrate the expression level and percentages of the top differentially expressed immune-related genes in various cell types in the TME of control and anti-Areg-antibody-treated mice.
